# Microbiota humanization drives human‐like metabolic and immune transcriptomic shifts in pigs

**DOI:** 10.1002/imo2.70034

**Published:** 2025-06-24

**Authors:** Zhaoqi Zhang, Yanan Xu, Kun Pang, Changhong Wu, Chenxu Zhao, Tong Lei, Jiayu Zhang, Tang Hai, Fangqing Zhao, Yong Zhao

**Affiliations:** ^1^ State Key Laboratory of Membrane Biology, Institute of Zoology Chinese Academy of Sciences Beijing China; ^2^ CAS Key Laboratory of Quantitative Engineering Biology, Shenzhen Institute of Synthetic Biology, Shenzhen Institute of Advanced Technology Chinese Academy of Sciences Shenzhen China; ^3^ Faculty of Synthetic Biology Shenzhen University of Advanced Technology China; ^4^ Department of Rheumatology and Immunology Peking University People's Hospital & Beijing Key Laboratory for Rheumatism Mechanism and Immune Diagnosis (BZ0135) Beijing China; ^5^ Institute of Zoology Chinese Academy of Sciences Beijing China; ^6^ Beijing Institute for Stem Cell and Regenerative Medicine Beijing China; ^7^ Beijing Farm Animal Research Center, Institute of Zoology Chinese Academy of Sciences Beijing China; ^8^ State Key Laboratory of Stem Cell and Reproductive Biology, Institute of Zoology Chinese Academy of Sciences Beijing China

**Keywords:** gut microbiota, human microbiota transplantation, humanized pigs, immune cells, metabolism, pigs

## Abstract

Pigs are increasingly recognized as promising candidates for clinical xenotransplantation and as large‐animal models for biomedical research; however, interspecies differences in gut microbiota, immune function, and metabolism remain major barriers. To address this, we established gut microbiota‐humanized (GMH) pigs by transplanting human fecal microbiota into antibiotic‐treated pigs. We systemically evaluated alterations in microbiota composition, serum metabolites, and immune cell profiles using integrated metagenomic, quasi‐targeted metabolomic and single‐cell transcriptomic (scRNA‐seq) analyses. Metagenomic profiling revealed a shift in the intestinal microbiota of GMH pigs toward a human‐like composition, characterized by enrichment of *Bacteroidia* and depletion of *Bacilli*. Metabolomic analysis showed that GMH pigs exhibited serum metabolite profiles more closely resembling those of humans. Among 423 detected serum metabolites, 136 that were lower in control pigs than in humans were upregulated in GMH pigs, whereas 79 that were elevated in control pigs decreased post‐transplantation. Notably, pathways related to tryptophan metabolism, bile acid biosynthesis, and fatty acid metabolism were enhanced in GMT pigs, while carbon‐related and glycolytic pathways were attenuated, indicating partial convergence toward human metabolic phenotype. Integration of microbial and metabolite data identified 20 and 33 metabolites associated with *Bacteroidia* and *Bacilli*, respectively. scRNA‐seq profiling of peripheral blood mononuclear cells demonstrated transcriptional and compositional remodeling of T cells, monocytes, and B cell subsets in GMH pigs. These findings demonstrated that human fecal microbiota can reshape both systemic metabolic and immune artitecture in pigs, offering a robust large‐animal platform for studying host‐microbiota interactions and advancing translational application in xenotransplantation and microbiome‐based therapeutics.

## Highlights

- Gut microbiota‐humanized pigs were successfully established via fecal microbiota transplantation.
- Human‐like remodeling of serum metabolome was achieved in humanized pigs.
- Transcriptional reprogramming of immune cell subsets in humanized pigs revealed shifts toward human‐like metabolic and biosynthetic activity.

## Graphical abstract

This study successfully established gut microbiota‐humanized pigs, achieved human‐like remodeling of the serum metabolome, and uncovered transcriptional alterations in immune cell subsets. These findings advance our understanding of how human gastrointestinal microorganisms affect host metabolism and the immune function in a porcine model.

## 1 INTRODUCTION

Pigs are considered highly promising organ donors for clinical xenotransplantation due to the physiological and anatomical similarity to humans [1, 2]. In addition to their relevance for organ donation, pigs represent a valuable non‐primate large animal model for preclinical research in human diseases, pharmacology, and transplantation [2]. Recent advances have enabled the transplantation of genetically engineered pig hearts and kidneys into brain‐dead human recipients, as well as into selected clinical cases, demonstrating the potential of genetic modification to overcome formidable immunological and physiological barriers. Despite this progress, further optimization is required to enhance immunological and physiological compatibility between pigs and humans, particularly in aspects such as cellular communications, metabolism, and microbiome.

Over the past two decades, the human gut microbiome has been gradually recognized as a critical determinant of host physiology, influencing health status and the pathogenesis of numerous diseases [3]. While the gut microbiota was traditionally described as a neutral symbiont (neither harmful nor beneficial), current understanding highlights a complex, often mutually beneficial relationship between host and microbes. The host supplies nutrients and a protected environment, while the microbiota contributes to digestion, immunity, and synthesis of essential metabolites, including vitamins B and K. Remarkably, up to 10% of circulating metabolites in mammalian are thought to originate from the gut microbiota [4]. Therefore, these microbial communities play important roles in regulating host metabolism, immunity, and neuroendocrine regulation [5, 6, 7].

Given the rapid growth and susceptibility to nutritional and metabolic regulation, pigs serve as a valuable system for investigating host‐microbiome interactions [1]. However, comparative studies have shown that porcine gut metagenome contains fewer predicted genes than the human gut metagenome and exhibits higher alpha diversity but lower beta diversity across taxonomic and functional categories, including gene content, genus‐level profiles, and Kyoto Encyclopedia of Genes and Genomes (KEGG) orthologies [8, 9]. Efforts to humanize the gut microbiota of animal models have largely been confined to mice and neonatal germ‐free piglets [9, 10, 11, 12]. Yet, the functional consequences of gut microbiota humanization in pigs on host serum metabolites, serum biochemical indices, as well as immune cell proportions and transcriptional profiles, remain poorly understood.

In this study, we established gut microbiota‐humanized pigs by transplanting human fecal microbiota into antibiotic‐treated juvenile pigs. We assessed the effects of microbial humanization on host serum metabolome and immune cell populations using quasi‐targeted metabolomics and single‐cell RNA sequencing (scRNA‐seq). Our findings provide new insights into how human gut microbiota influences porcine metabolism and immunity. This study offers a foundational step toward the development of gut microbiota‐humanized pig models for human biomedical research, and potentially, toward the generation of optimized porcine donors for clinical xenotransplantation following further validation of functional benefits.

## 2 RESULTS

### 2.1 Establishment of humanized gut microbiota in pigs

**Figure 1:**
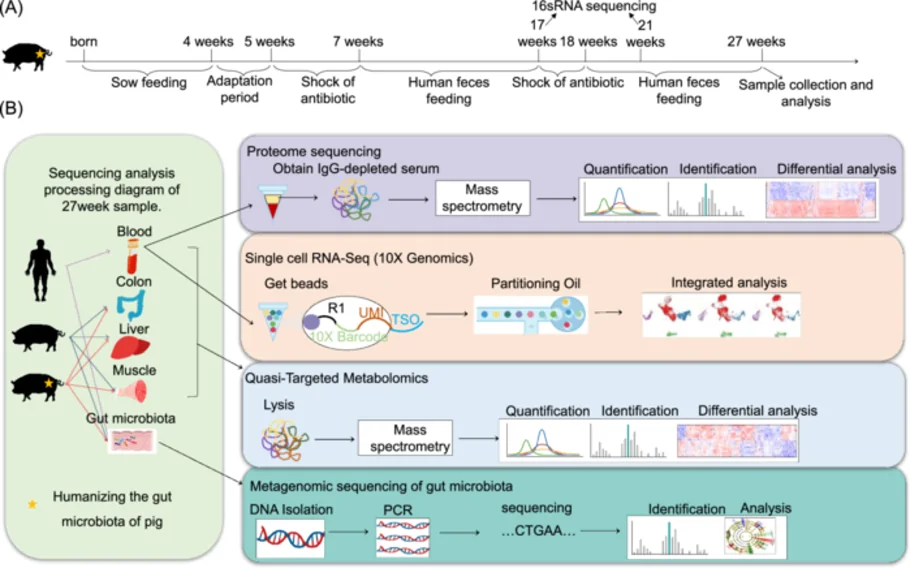
Construction of the human microbiota transplanted pig. (A) The establishment and analysis procedures of humanized pigs. Weeks, age in weeks of pigs under different treatments. (B) Schematic diagram of samples and analysis procedures in this study. We collected feces from four people and mixed them to feed pigs. Sample number of gut microbiota for metagenomic sequencing: Four human feces donors, six control pigs, and six human feces‐transplanted pigs. Sample number of 16sRNA sequencing: Four human feces donors, six control pigs, and six human feces‐transplanted pigs. Sample number of proteome sequencing: Five human samples (four feces donors and one additional sample), six pigs, and six human feces‐transplanted pigs. Sample number of 10× Genomics scRNA‐Seq: Two human feces donors, two control pigs, and twohuman feces‐transplanted pigs. Sample number of serum Quasi‐Targeted Metabolomics: Five humans, six control pigs, and six human feces‐transplanted pigs. Sample number of colon, liver, and muscle Quasi‐Targeted Metabolomics: Six control pigs, and six human feces‐transplanted pigs. scRNA‐Seq, single‐cell RNA sequencing.

To construct pigs with a human‐like gut microbiota, we first collected feces from four healthy donors in accordance with the volunteer screening criteria (Table S1). Pigs in both the experimental and control groups were weaned at 4 weeks of age and received broad‐spectrum antibiotics, followed by either fecal microbiota transplantation (FMT), as detailed in Table S2. The experimental group, hereafter referred to as GMH pigs, was provided with donor feces from weeks 7 to 17 and again from weeks 18 to 27. All animals underwent antibiotics treatment during weeks 5–7 and 17–18 to reduce endogenous microbiota before FMT (Figure 1A).

To assess the extent of microbial humanization, we performed 16s RNA sequencing on fecal samples collected from control and GMH pigs at weeks 17 and 21, using donor fecal samples as the human reference. At week 17, both groups displayed comparable microbiota profiles. However, by week 21, after the second antibiotic course, the fecal microbiota of GMH pigs shifted significantly toward a human‐like composition, diverging from controls (Figure S1).

**Figure 2:**
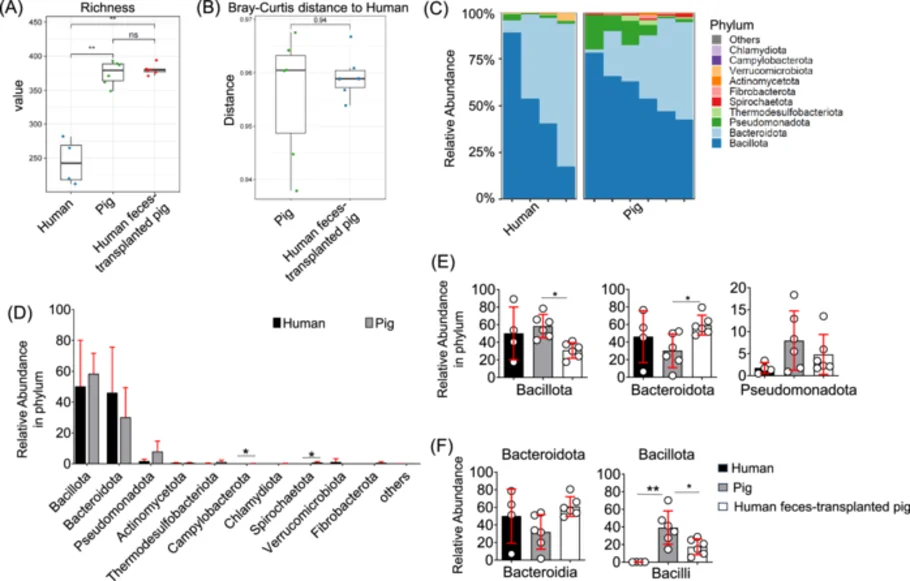
Composition changes of the gut microbiome in the humanized pigs. (A) Statistical chart of gut flora richness analysis. (B) Bray–Curtis distance to human analysis between control pigs and human feces‐transplanted pigs. (C) Phylum‐level sample composition. (D) Statistical diagram of the differences in phylum‐level composition of intestinal flora between humans and pigs. (E) Statistical chart of phylum‐level intestinal flora with humanization trend. (F) Class‐level bacterial communities with humanization trends under Bacteroidota and Bacillota, respectively. Sample number of intestinal flora sequencing: Four human feces donors, six control pigs, and six human feces‐transplanted pigs. Data in human, Pig, and humanized pig samples are presented as mean ± SD. (*) *p* < 0.05, (**) *p* < 0.01, data were examined using one‐way ANOVA, with Bonferroni correction.

To enable integrated cross‐omics comparisons, we collected plasma, peripheral blood mononuclear cells (PBMCs), intestinal tissue, liver, muscle and fecal samples from both groups at 27 weeks of age. These samples were subjected to metagenomic sequencing, quasi‐targeted metabolomics, quantitative proteomics, and scRNA‐seq (Figure 1B and Table S3). Consistent with previous reports [13], control pigs harbored a much richer gut microbiota than that of humans. Analysis of metagenomic data from donors, control pigs, and GMH pigs using weighted UniFrac distance revealed that GMH pigs' microbiota composition was significantly closer to human donors' than to control pigs' [14] (Table S4), although overall microbial richness and beta‐diversity in both pig groups remained greater than in human donors (Figure 2A,B).

At the phylum level, significant differences were observed between humans and pigs in *Campylobacterota* and *Spirochaetota* (*p* < 0.05, Figure 2C,D). Additionally, the relative abundances of *Bacillota* and *Bacteroidota* differed significantly between control and GMH pigs (*p* < 0.05, Figure 2E). Class‐level comparisons of the three altered bacterial phyla revealed that only *Bacteroidia* and *Bacilli* in GMH pigs approached levels observed in humans and diverged from control pigs (Figures 2F and S2). Specifically, the GMH group showed an increased proportion of *Bacteroidia* and a markedly decreased proportion of *Bacilli* compared with control pigs, indicating a more human‐like microbial composition (Figure 2F and Figures S2, S3).

Collectively, these results demonstrate that FMT following antibiotic conditioning can effectively humanize the gut microbiota of pigs, establishing GMH pig as a valuable large‐animal model for translational studies.

### 2.2 Humanized gut microbiota alters the serum metabolite profile in pig

To assess the physiological impact of gut microbiota humanization, we measured body weight and body temperature in both antibiotic‐treated control pigs and GMH pigs weekly following interventions. No significant differences were observed between the two groups in either parameter (Figure S4A, S4B). Likewise, measurements of blood pressure and heart rate at weeks 20 and 23 showed no significant differences (Figure S4C, S4D). These results suggested that the transplantation of human feces did not impair overall health or growth of the pigs.

**Figure 3:**
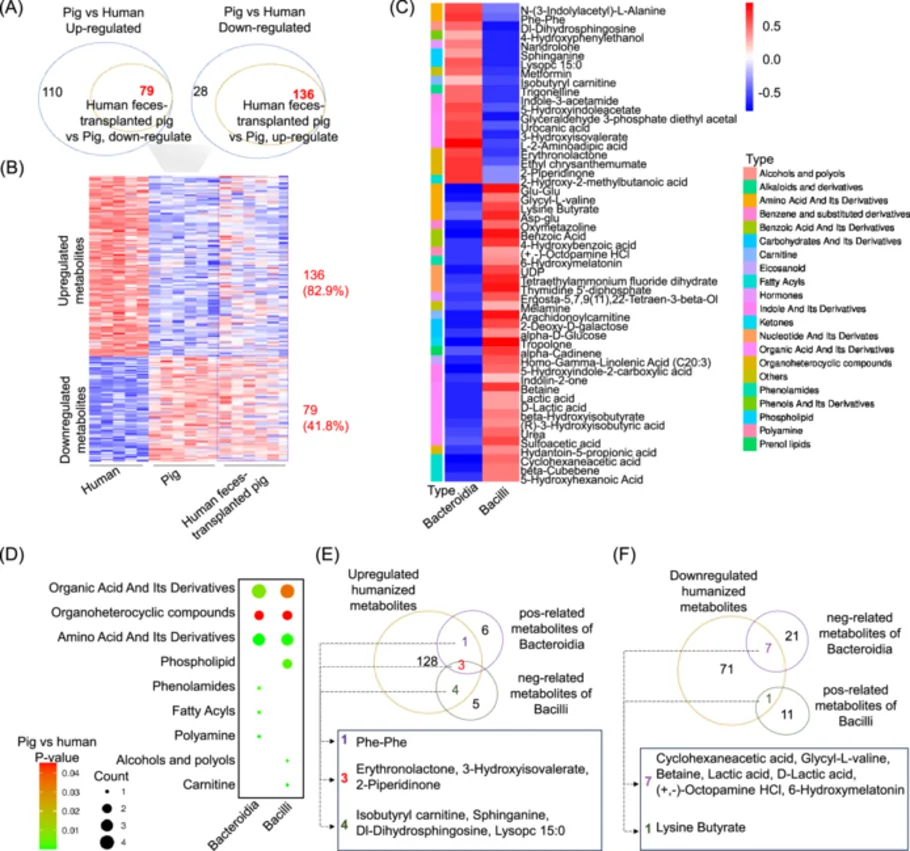
Effects of intestinal flora on serum metabolic levels in human feces‐transplanted pigs. Venn diagram (A) and heatmap (B) of serum metabolites with humanization trends. (C) Heatmap of the correlation between intestinal flora and metabolite content. Red indicates positive correlation, and blue indicates negative correlation. (D) Bubble chart of metabolite function analysis. Venn diagram of upregulated (E) and downregulated (F) humanized metabolites regulated by *Bacteroidia* and *Bacilli*. Sample number of intestinal flora sequencing samples and serum metabolome sequencing: Five human feces donors, six control pigs, and six human feces‐transplanted pigs.

Microbial communities are known to influence host physiology through the production of a broad spectrum of metabolites [15]. To determine whether human FMT could shift host systemic metabolism toward a human‐like state, we performed quasi‐targeted metabolomics on serum collected at 27 weeks from control pigs (*n* = 6) and GMH pigs (*n* = 6) (Figure S5A). For cross‐species comparison, human serum samples (*n* = 5) were collected independently from donors. Across all samples, a total of 423 metabolites were identified (Figure S5B). Comparative analysis between human and control pigs revealed significant interspecies differences. Control pigs exhibited lower levels of metabolites involved in tryptophan, bile acid, sphingomyelin, and linoleic acid metabolism (Figure S6A), and higher levels of metabolites associated with arginine‐proline metabolism, pyruvate metabolism, pentose phosphate metabolism, and other carbon‐related pathways (Figure S6B). Notably, human FMT substantially reduced these differences, shifting the porcine serum metabolome toward a human‐like profile. Among the 189 metabolites that were elevated in control pigs versus humans, 79 (41.8%) were reduced in GMH pigs (Figure 3A,B). Conversely, of the 164 metabolites that were lower in pigs than humans, 136 (82.9%) were increased post‐transplantation (Figure 3A,B). Pathway enrichment analysis indicated that pyruvate, pentose phosphate, and carbon‐related metabolism pathways, which were enriched in control pigs, were significantly reduced in GMH pigs (Figures S7 and S8A). In contrast, tryptophan, bile acid, and fatty acid metabolic pathways, which were underrepresented in control pigs, were upregulated in GMH pigs, trending toward human‐like levels (Figures S7 and S8B).

These metabolic shifts were further supported by clinical blood biochemical analysis, which showed lower glucose and higher triglyceride (TG) concentrations in the GMH pigs compared to controls, consistent with the observed metabolomic trends (Figure S9). In addition, tissue metabolomics showed directional changes: most metabolite changes observed in serum were also reflected in the colon, liver, and muscle tissues (Figure 3A and Figures S10A, S10B). In GMH pigs, the upregulated serum metabolites were predominantly consistent with those found in muscle, while the downregulated metabolites were more closely aligned with those in the colon (Figure S10C, S10D). By contrast, global serum proteomic analysis showed no significant difference between GMH and control groups (Figure S11), suggesting that the observed metabolic effects were not accompanied by circulating protein levels.

To further understand microbiota‐metabolite interactions, we integrated metagenomic and metabolomic datasets across humans, control pigs, and GMH pigs. Correlation analysis identified 20 metabolites positively associated with the abundance of *Bacteroidia*, and 33 associated with *Bacilli* (Figure 3C). These metabolites span multiple chemical classes, including organic acid, organoheterocyclic compounds, amino acid, phospholipid, phenolamides, and fatty acyls (Figure 3D). Given that GMH pigs showed elevated *Bacteroidia* and reduced *Bacilli* levels compared to controls (Figure 2F), we infer these taxa contributed to the observed metabolic shifts. Specifically, erythronolactone, 3‐hydroxyisovalerate, and 2‐piperidinone were associated with both classes (Figure 3E). Other metabolites, such as Phe‐Phe, betaine, lactic acid, d‐lactic acid, cyclohexaneacetic acid, glycyl‐l‐valine, (+/−)‐octopamine HCl, and 6‐hydroxymelatonin, were primarily linked to *Bacteroidia*, while isobutyryl carnitine, sphinganine, Dl‐dihydrosphingosine, lysoPC 15:0 and lysine butyrate were more strongly associated with *Bacilli* (Figure 3E,F).

Taken together, these results demonstrated that human FMT drives significant remodeling of the porcine serum metabolome in GMH pigs. These changes are closely tied to alterations in gut microbiota composition and shift the systemic metabolic e profile toward a human‐like state, supporting the successful establishment of a microbially and metabolically humanized pig model.

### 2.3 Single cell transcriptome atlas of PBMCs in humans, pigs, and GMH pigs

**Figure 4:**
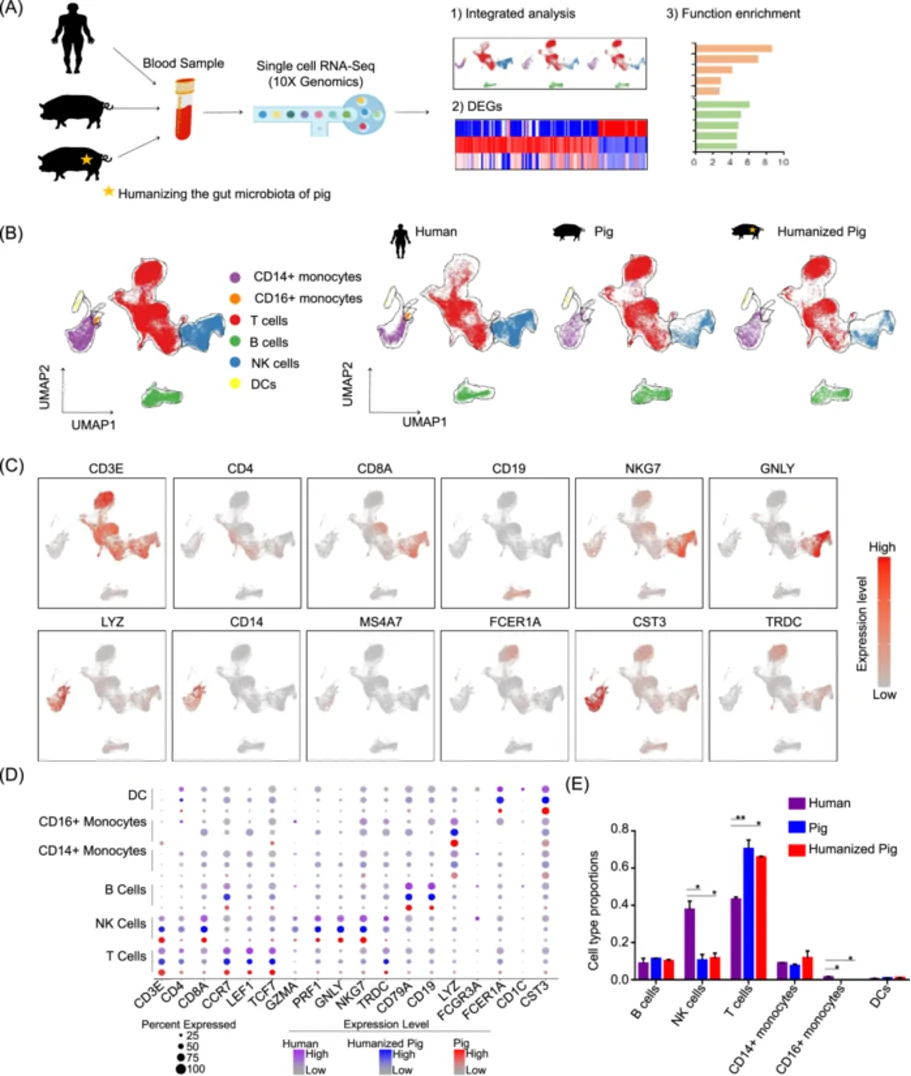
Construction of human‐pig‐humanized pig integrated scRNA‐seq from PBMCs. (A) Schematic diagram of samples and analysis procedures in this study. (B) Uniform Manifold Approximation and Projection (UMAP) visualization of human‐pig‐humanized pig integrated clustering. And the UMAP visualization of separated human cells, pig cells, and humanized pig cells for all cells. Each dot represents a cell, which is colored according to cell type. (C) Immune cell markers presented by UMAP feature plot, with UMAP plot color indicating the level of gene expression for all cells. (D) Bubble plot showing marker expression levels in human, pig and humanized pig. Purple represents the expression level in human, blue represents expression level in pig, and red represents expression level in humanized pig. (E) Statistical chart of the proportions of human‐pig‐humanized pig cell subpopulations. Sample number of PBMCs for scRNA sequencing: Two humans, two pigs, and two human feces‐transplanted pigs. Data in human, pig, and humanized pig samples are presented as mean ± SD. (*) *p* < 0.05, (**) *p* < 0.01, data were examined using one‐way ANOVA, with Bonferroni correction. scRNA‐Seq, single‐cell RNA sequencing.

To compare immune cell transcriptomic profiles at single‐cell resolution across species, we performed scRNA‐seq on PBMCs from humans, control pigs, and GMH pigs. PBMCs were isolated from two healthy human donors, two control pigs (27 weeks of age), and two GMH pigs (27 weeks of age). Single‐cell libraries were prepared and sequenced using the 10× Genomics platform (Figure 1A). Using a previously validated strategy [2], we aligned transcriptomes based on 14,377 homologous genes. In total, we profiled 24,090 cells from humans, 20,402 from control pigs, and 15,837 from GMH pigs (Table S5 and Figure S12). The three datasets were integrated and subjected to unsupervised clustering, initially identifying 26 distinct cell populations (Figure S13). Clusters exhibiting low expression of *PTPRC* (CD45) or enriched in platelet‐associated genes (clusters 14, 20, 21, and 23) were excluded from downstream analysis (Figure S14) [2]. After re‐clustering, we resolved 31 transcriptionally distinct immune cell populations (Figure S15). Annotation was guided by canonical human immune cell markers, resulting in six major immune lineages visualized by Uniform Manifold Approximation and Projection (UMAP), including T cells, nature killer (NK) cells, B cells, CD14^+^ monocytes, CD16^+^ monocytes, and dendritic cells (DCs) (Figure 4A,B and Table S6). Cell‐type identities were supported by conserved expression of lineage‐defining genes across species (Figure 4C and Figure S16). These included *CD8A*, *CD4*, *IL7R*, and *CD3E* for T cells; *NKG7* and *GNLY* for NK cells; *CD19*, *CD79B*, and *MS4A1* for B cells; *LYZ* and *CD14* for CD14^+^ monocytes; *MS4A7* and *LYZ* for CD16^+^ monocytes; and *FCER1A* and *CST3* for DCs. Expression levels of subset‐specific transcriptional regulators and surface molecules, including *CD3E, CD4, CCR7, LEF1, TCF7, GNLY, NKG7, CD79A, LYZ*, and *CST3*, were well conserved between humans and pigs, validating cross‐species annotations (Figure 4D).

To refine classification, we applied updated human‐pig ortholog mappings and leveraged well‐defined human PBMC transcriptomic references to infer the distribution of porcine immune subsets. This cross‐species framework enabled comparative quantification of immune cell proportions across groups (Figure 4E). Relative to humans, pigs exhibited higher proportions of T cells, but lower frequencies of NK cells and CD16^+^ monocytes (Figure 4E). Notably, GMH pigs showed a cellular composition more similar to humans, especially in T cells and CD14^+^ monocytes (Figure 4E), suggesting a microbiota‐driven modulation of immune composition. These results establish a high‐resolution single‐cell atlas of PBMCs across humans, pigs, and GMH pigs.

### 2.4 Single‐cell dissection of T cell subsets in humans, pigs, and GMH pigs

**Figure 5:**
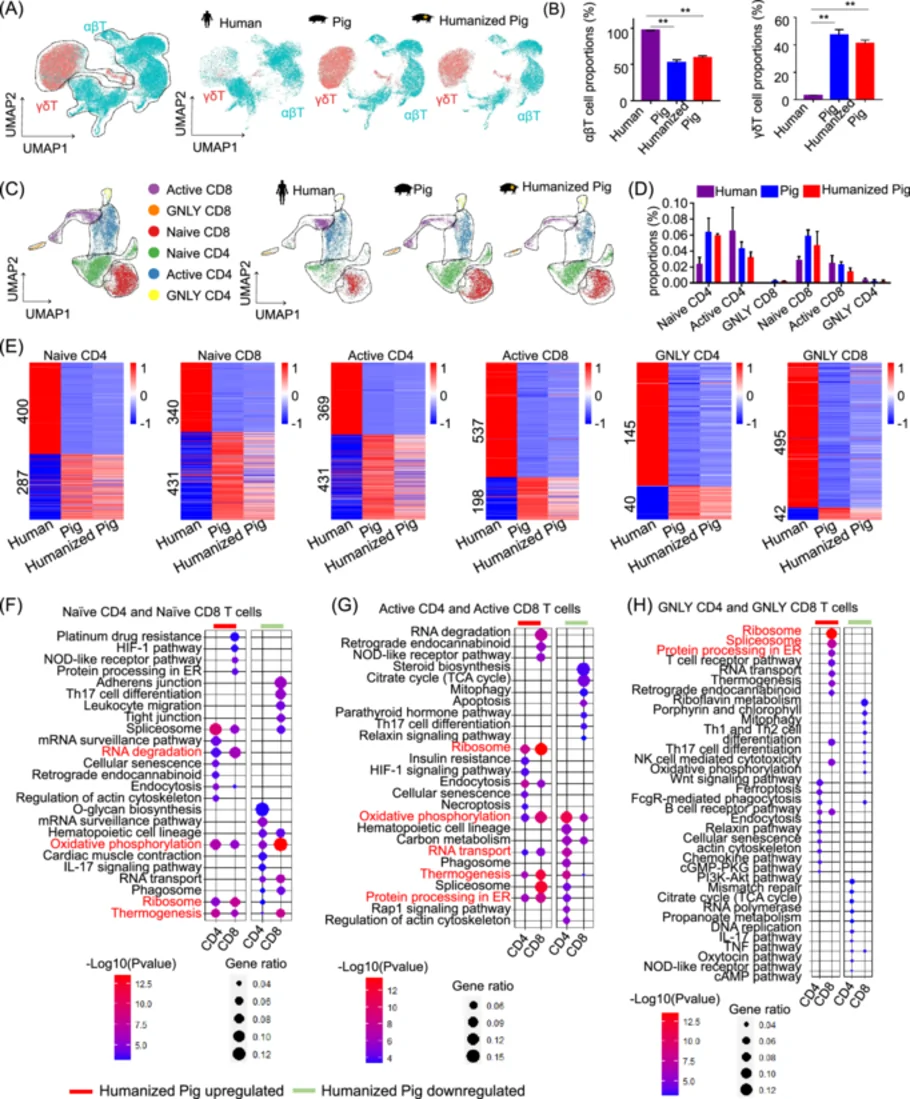
Dissection and reclustering of T cells from integrated human‐pig‐humanized pig scRNA‐seq data. (A) Uniform Manifold Approximation and Projection (UMAP) visualization of human‐pig‐humanized pig integrated clustering of αβ T cells and γδ T cells. Pink labeled γδ T cells and blue labeled αβ T cells. (B) The relative cell proportions (%) of αβ T cells and γδ T cells in humans, pigs and humanized pigs, respectively. (C) UMAP visualization of human‐pig‐humanized pig integrated clustering of αβ T cells. (D) The relative cell proportions (%) of αβ T cell subsets in humans, pigs, and humanized pigs, respectively. (E) Genes with humanization trends in humans, pigs, and humanized pigs in various T cell subsets, as presented by a heatmap. KEGG enrichment of genes with humanization trends in humans, pigs, and humanized pigs in naive T cells (F), active T cells (G), and GNLY + T cells (H). Sample number of PBMCs for scRNA sequencing: Two humans, two pigs, and two human feces‐transplanted pigs. Data in human, pig, and humanized pig samples are presented as mean ± SD. (**) *p* < 0.01, data were examined using one‐way ANOVA, with Bonferroni correction. KEGG, Kyoto Encyclopedia of Genes and Genomes. scRNA‐Seq, single‐cell RNA sequencing.

T cells constitute a major component of PBMCs. To dissect T cell heterogeneity across species, we extracted T cells from the integrated scRNA‐seq data set for sub‐clustering (Figure S17). Based on established human T cell gene signatures, we identified two major T cell lineages: αβT cells and γδT cells (Figure 5A and Figure S18). Quantification of these subsets revealed that αβT cells dominated in human PBMCs, while pig T cells contained a relatively high proportion of γδT cells (Figure 5B). This lineage distribution remained largely unchanged in GMH pigs (Figure 5B).

Next, we further sub‐classified γδT cells into CD2^−^γδT cells and CD2^+^γδT cells based on their gene expression signatures (Figures S19 and S20A). In GMH pigs, the relative abundance of CD2^−^ γδT cells was reduced, suggesting a microbiota‐dependent modulation of this subset (Figure S20B). Functional enrichment analysis showed that CD2^−^ γδT cells were enriched in pathways associated with tight junctions, apoptosis and phagosome activity (Figure S20C, S20D). Furthermore, this subset exhibited high expression of genes related to cell proliferation and cell cycle (FOS, JUN, RHEX, and SELL), indicating that CD2^−^ γδT cells may represent a precursor‐biased population (Figure S20E).

To investigate the effects of gut microbiota humanization on αβT cell states in detail, we extracted αβT cells from the integrated data set for high‐resolution clustering (Figure 5A). Based on canonical markers, we annotated 6 distinct αβT cell subsets across species: naive CD4^+^ T cells (*CCR7*, *IL7R*, and *CD4*), naive CD8^+^ T cells (*CCR7*, *IL7R*, and *CD8A*), activated CD4^+^ T cells (*CD4* and *S100A4*), GNLY^+^CD4^+^ T cells (*GNLY* and *CD4*), GNLY^+^CD8^+^ T cells (*GNLY* and *CD8A*) and activated CD8^+^ T cells (*CD8A* and *S100A4*) (Figure 5C and Table S7). Although the relative proportions of these αβT cells were not significantly altered in GMH pigs (Figure 5D), transcriptomic comparisons revealed human‐like expression patterns in multiple subsets. Specifically, naive and activated T cell populations had the greatest transcriptional changes following transplantation, with 687, 771, 800 and 735 genes showing human‐like expression in naive CD4^+^, naive CD8^+^, activated CD4^+^ T, and activated CD8^+^ T cells, respectively. In contrast, GNLY^+^ T cells, representing terminally differentiated cytotoxic subsets, showed less changes (Figure 5E).

To further explore functional changes, we performed KEGG pathway enrichment analysis of genes expressed in GMH pig T cells that showed expression patterns consistent with human cells. In naive CD4+ and CD8 + T cells, the upregulated genes were mainly enriched in biosynthetic and metabolic pathways, including spliceosome assembly, RNA degradation, oxidative phosphorylation, ribosome biogenesis, and thermogenesis. Conversely, pathways related to adherent junctions, Th17 cell differentiation, and leukocyte migration were downregulated in naive CD8^+^ T cells (Figure 5F). Similarly, the upregulated genes in activated CD4+ and CD8+ T cells were enriched in translational and metabolic processes such as ribosome, oxidative phosphorylation, RNA transport, thermogenesis, and protein processing in endoplasmic reticulum (ER) (Figure 5G). GNLY^+^ CD8^+^ T cells also displayed enrichment for ribosomal and ER protein processing pathways (Figure 5H).

In summary, these findings indicated that human fecal transplantation induces remodeling of T cell subsets in pigs. While overall subset proportions remained relatively stable, gene expression programs in naive and activated T cells notably shifted toward human‐like patterns, particularly in metabolic and biosynthetic processes.

### 2.5 Single‐cell dissection of myeloid immune cell populations in humans, pigs, and GMH pigs

**Figure 6:**
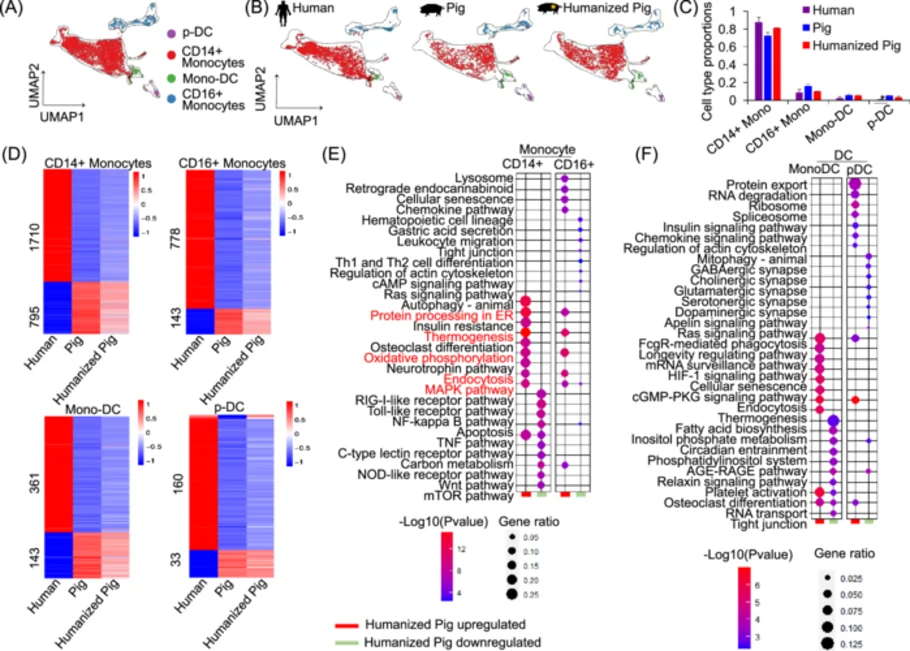
Dissection and reclustering of myeloid cells from integrated human‐pig‐humanized pig scRNA‐seq data. (A) Uniform Manifold Approximation and Projection (UMAP) visualization of human‐pig‐humanized pig integrated clustering of myeloid cells. (B) UMAP visualization of separated human cells, pig cells and humanized pig cells for myeloid cells. Different colors represent different clusters. (C) The relative cell proportions (%) of myeloid cell subsets in humans, pigs and humanized pigs, respectively. (D) Genes with humanization trends in humans, pigs, and humanized pigs in various myeloid cell subsets, as presented by a heatmap. KEGG enrichment of genes with humanization trends in humans, pigs, and humanized pigs in monocytes (E) and DCs (F). Sample number of PBMCs for scRNA sequencing: Two humans, two pigs, and two human feces‐transplanted pigs. Data in human, pig, and humanized pig samples are presented as mean ± SD. KEGG, Kyoto Encyclopedia of Genes and Genomes. scRNA‐Seq, single‐cell RNA sequencing.

To investigate the composition and transcriptional profiles of circulating myeloid immune cells across humans, pigs, and microbiota‐humanized pigs, we extracted and integrated PBMC‐derived myeloid cells from all three groups. Unsupervised clustering identified 4 major populations, including CD14^+^ monocytes, CD16^+^ monocytes, monocyte‐derived dendritic cells (mono‐DCs), and plasmacytoid dendritic cells (p‐DCs), based on canonical human marker genes (Figure 6A,B and Figure S21). Highly expressed marker genes defining each subset were shown in Figure S22 and Table S8. Among these, CD14^+^ monocytes constituted the predominant myeloid subset across humans, pigs, and GMH pigs (Figure 6C). Interestingly, both control and GMH pigs exhibited a distinct cluster transcriptionally resembling human CD16^+^ monocytes, this population displayed low expression of *CD16* (*FCGR3A*) itself (Figure S21C), suggesting a species‐ or condition‐specific divergence despite shared gene expression features.

To systematically assess conservation across these myeloid subsets, we assessed the expression of established human myeloid marker genes within the corresponding cell types from control pigs and GMH pigs. Overall, marker gene expression patterns were well conserved across species and conditions. For example, CD14^+^ monocytes consistently expressed *CD14, S100A8, C5AR1, S100A9*, and *S100A12*; mono‐DCs expressed *PKIB, FCER1A, ITGB7, HLA‐DOA*, and *HLA‐DQB1*; and p‐DCs expressed *FAM129C, ITM2C, CXCR3, PLAC8*, and *VEGFB* (Figure S21D). These results validated the integration and annotation strategy across species and experimental groups.

To evaluate the effects of human microbiota colonization on myeloid cell function in pigs, we performed differential gene expression analysis comparing control pigs, GMH pigs, and humans. We focused on genes that were (1) more highly expressed in control pigs relative to humans but downregulated in humanized pigs, or (2) under‐expressed in pigs relative to humans but upregulated after humanization (Figure 6D). Among the four cell types analyzed, CD14^+^ and CD16^+^ monocytes displayed the greatest degrees of transcriptional reprogramming following human fecal transplantation, with 2505 and 921 differentially expressed genes (DEGs), respectively. In contrast, mono‐DCs (504 DEGs) and p‐DCs (193 DEGs) showed more modest changes (Figure 6D), indicating a heightened responsiveness of monocyte subset to gut microbiota modulation.

KEGG pathway enrichment analysis of DEGs with human‐like expression patterns revealed consistent biological themes. In CD14^+^ and CD16^+^ monocytes, genes upregulated following fecal transplantation were enriched in pathways related to protein processing in the ER, thermogenesis, oxidative phosphorylation, endocytosis, and MAPK signaling (Figure 6E), which paralleled transcriptional shifts previously observed in T cells. In mono‐DCs, upregulated genes were mainly enriched in Fcγ receptor (FcγR)‐mediated phagocytosis, mRNA surveillance, and longevity‐regulating pathway, while downregulated genes were associated with fatty acid biosynthesis, inositol phosphate metabolism and circadian entrainment (Figure 6F). In p‐DCs, upregulated genes were enriched in pathways such as protein export, RNA degradation, ribosome, and endocytosis (Figure 6F). Unexpectedly, we observed significantly downregulation of synapse‐related pathway in p‐DCs from GMH pigs. Further gene‐level analysis identified *GNG2* and *GNG10* (Table S9), key components of the Ras signaling pathway, as the primary contributors to this enrichment. These findings suggested that Ras‐mediated signaling may be selectively dampened in pDCs in pigs following human microbiota colonization.

Taken together, these data demonstrated that transplantation of human gut microbiota induces broad and cell‐type ^+^specific transcriptional reprogramming of circulating myeloid cells in pigs. Monocytes, in particular, exhibited strong transcriptomic shifts toward human‐like profiles, indicating the capacity of the gut microbiota to modulate systemic innate immune function.

### 2.6 Human FMT modulates the composition and transcriptional profile of pig B cells

**Figure 7:**
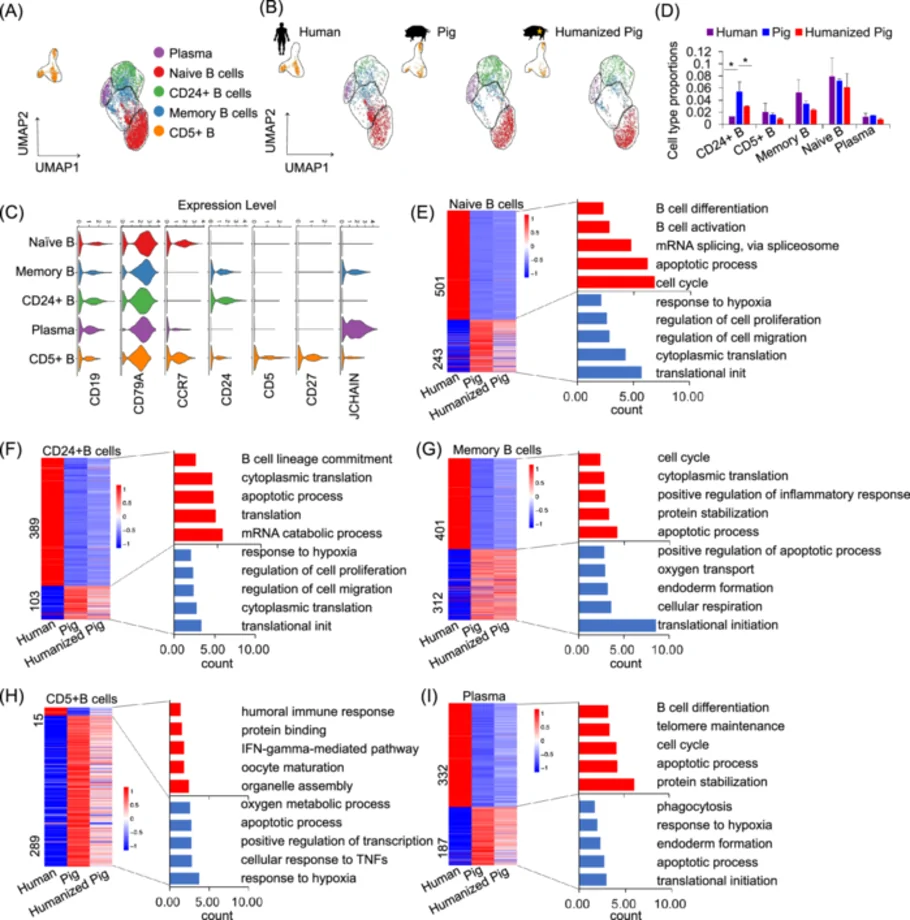
Dissection and reclustering of B cells from integrated human‐pig‐humanized pig scRNA‐seq data. (A) Uniform Manifold Approximation and Projection (UMAP) visualization of human‐pig‐humanized pig integrated clustering of B cells. (B) UMAP visualization of separated human cells, pig cells and humanized pig cells for B cells. Different colors represent different clusters. (C) Specific immune cell markers presented as violin plots, with colors labeling different clusters. (D) The relative cell proportions (%) of B cell subsets in humans, pigs and humanized pigs, respectively. GO enrichment of genes with humanization trends in humans, pigs, and humanized pigs in naive B cells (E), CD24^+^ B cells (F), memory B cells (G), CD5^+^ B cells (H) and plasma cells (I). Sample number of PBMCs for scRNA sequencing: Two humans, two pigs, and two human feces‐transplanted pigs. Data in human, pig, and humanized pig samples are presented as mean ± SD. (*) *p* < 0.01, data were examined using one‐way ANOVA, with Bonferroni correction. GO, Gene Ontology; scRNA‐Seq, single‐cell RNA sequencing.

Unlike T cells and monocytes, the overall proportion of B cells in PBMCs remained relatively stable across GMH pigs, control pigs and humans, with no significant difference observed (Figure 4E). To investigate whether human FMT induces phenotypic and transcriptional changes in pig B cells, we extracted B cells from the integrated scRNA‐seq data set comparing humans, control pigs, and GMH pigs. Unsupervised clustering identified 5 B cell subpopulations: naïve B cells (*CD19* ^*+*^, *CD79A* ^*+*^, *CD2* ^*+*^, and *CCR7* ^*+*^), memory B cells (*CD19* ^*+*^, *CD79A* ^*+*^, *CD2^−^*, and *CD27* ^*+*^), CD24^+^ B cells (*CD19* ^*+*^, *CD79A* ^*+*^, and *CD24* ^*+*^), CD5^+^ B cells (*CD19* ^*+*^, *CD79A* ^*+*^, and *CD5* ^+^) and plasma cells (*CD19* ^*+*^, *CD79A* ^*+*^, *CD2^−^*, and *JCHAIN* ^+^), as annotated based on canonical human B cell marker genes (Figure 7A,B and Figure S23) [16]. Signature gene expression for each subset is shown in Figure 7C, Figure S24, and Table S10.

Among these subsets, CD24^+^ B cells exhibited the most notable compositional shift. CD24 is well‐characterized marker of immature B cells and plays a role in B cell maturation and signaling [17]. Studies have shown that CD24^+^ B cells in pigs may be involved in genes regulating BCR signaling and cell proliferation regulation. Consistent with prior reports, CD24^+^ B cells were present at higher frequencies in control pigs compared to humans. However, following human FMT, the proportion of CD24^+^ B cells in pigs was significantly reduced, approaching levels observed in humans (Figure 7D). This suggests that gut microbiota composition may influence B cell developmental states.

To assess transcriptional response to FMT, we performed differential gene expression analysis across the 5 B cell subsets in humans, control pigs and GMH pigs, using the human gene expression profiles as reference. We identified a set of genes within na ive B cells, memory B cells, CD24⁺ B cells, CD5⁺ B cells, and plasma cells that exhibited “human‐like” expression patterns following transplantation (Figure 7E–I). Gene Ontology (GO) enrichment analysis of the DEGs further revealed functional implications of this shift. Genes that were underexpressed in control pigs compared to humans, but upregulated in GMH pigs, were enriched in pathways related to cell cycle progression, survival, and B cell development. Conversely, genes downregulated in GMH pigs were associated with response to hypoxia, cell proliferation, and cell migration. These patterns suggested that gut microbiota‐humanization not only alters the distribution of B cell subtypes but also modulates core biological processes associated with B cell maturation and function in pigs.

Taken together, these results demonstrated that human FMT significantly reshapes both the composition and gene expression of porcine B cells, promoting a shift toward a more human‐like immune phenotype.

## 3 DISCUSSION

The gut microbiota plays a vital role in regulating human health and disease susceptibility [18]. Although rodent models, particularly germ‐free mice and rats, have been widely employed to investigate human gut microbial composition and function [19, 20, 21], their translational relevance is limited due to substantial interspecies differences. To address this, we developed a porcine model of humanized gut microbiota by transplanting human fecal samples into antibiotic‐treated pigs. This model provides a promising alternative for preclinical research and potentially for clinical applications, including xenotransplantation. While germ‐free pigs have been used in previous humanization studies [22, 23, 24], practical constraints such as housing and sterility requirements limited their scalability. We therefore adapted previously reported methods [25]: recipient pigs were pretreated with broad‐spectrum antibiotics to deplete the endogenous gut microbiota, followed by FMT using cryopreserved human feces. Although fecal sample were stored at −80°C immediately after collection, the oral administration route may have compromised the viability of obligate anaerobes [26]. Furthermore, the absence of gnotobiotic environment likely permitted interspecies microbial competition, which may have impacted colonization efficiency.

Despite these limitations, our analyses demonstrated successful microbial engraftment and metabolic remodeling in the recipient pigs. Metagenomic profiling revealed the enrichment of *Bacilli* and *Bacteroidia* in humanized pigs, which are also implicated in microbiota‐humanized mouse models [27]. We identified 136 metabolites that were significantly upregulated in humanized pigs relative to controls, resembling human profiles, and 79 metabolites that were downregulated toward human‐like levels. Correlation analysis further revealed that 20 metabolites that were positively associated with *Bacteroidia*, and 33 metabolites with *Bacilli*. Notably, three metabolites (Erythronolactone, 3‐Hydroxyisovalerate, 2‐Piperidinone) were strongly correlated with both *Bacteroidia* and *Bacilli* abundance, underscoring a potential microbiota‐metabolite axis influenced by humanization.

To determine the systemic impact of microbiota humanization on host immunity, we performed integrative scRNA‐seq of PBMCs from humans, pig, and GMH pigs. By updating the human‐pig orthologous gene annotations and applying canonical immune cell markers [28, 29, 30], we accurately defined major immune subsets and their relative abundances. Compared to humans, pigs exhibited a higher proportion of T cells, particularly γδT cells. Fecal transplantation reduced total T cell abundance and specifically decreased the proportion of CD2^‐^ γδT cells in pigs, partially aligning the profile with that of humans. Although αβT cell proportions remained relatively unchanged, transcriptomic analysis revealed that naive and effector T cell populations (naive CD4^+^ T cells, naive CD8^+^ T cells, activated CD4^+^ T cells, GNLY^+^CD4^+^ T cells, GNLY^+^CD8^+^ T cells, and activated CD8^+^ T cells) displayed partial restoration of human‐like gene expression patterns in GMH pigs. Genes upregulated in pig T cells after fecal transplantation were mainly enriched in biosynthetic and metabolic pathways, such as spliceosome, RNA degradation, oxidative phosphorylation, ribosome biogenesis, and thermogenesis, highlighting enhanced metabolic programming in T cells of recipient pigs in response to the humanization of gut microbiota.

Similar trends were observed in monocyte populations. FMT increased the abundance of CD14^+^ monocytes, which were underrepresented in control pigs. CD14^+^ and CD16^+^ monocytes showed the most transcriptional responsiveness, with 2505 and 921 DEGs, respectively, between gut microbiota‐humanized and control pigs. These genes were enriched in pathways related to protein processing in the ER, thermogenesis, oxidative phosphorylation, endocytosis, and MAPK signaling, further indicating that enhanced metabolic activation following human microbiota transplantation.

B cell compartmentalization also reflected humanization effects. We identified five distinct B cell subsets from the integrated human, pig, and gut‐microbiota‐humanized pig scRNA‐seq data: naive B cells (CD19^+^, CD79A^+^, CD2^+^ and CCR7^+^), memory B cells (CD19^+^, CD79A^+^, CD2^−^, and CD27^+^), CD24^+^ B cells (CD19^+^, CD79A^+^, and CD24^+^), CD5^+^ B cells (CD19^+^, CD79A^+^, and CD5^+^) and plasma cells (CD19^+^, CD79A^+^, CD2^−^, and JCHAIN^+^). Comparative analysis of subset proportions revealed that CD24^+^ B cells, higher in control pigs relative to humans, were significantly reduced in GMH pigs, exhibiting a shift toward the human B cell profile. Transcriptomic analysis further demonstrated that naïve and memory B cells were particularly responsive to gut microbiota humanization, with 744 and 713 DEGs, respectively, in humanized versus control pigs. CD24^+^ B cells in human are known to play regulatory functions in immune‐mediated pathologies and suppress excessive immune responses [31, 32]. In pigs, CD24^+^ B cells have been characterized as atypical memory B cells, likely arising from repeated BCR stimulation, and are implicated in antiviral humoral immunity and inflammatory regulation [33]. Our previous research found that pig CD24^+^ B cells share transcriptomic features with human T‐bet^+^ B cells [2], which are involved in sustained immune response to pathogens. Together, these findings supported the conclusion that gut microbiota humanization regulates both the proportion and transcriptional phenotype of CD24^+^ B cells, making the porcine B cell landscape more closely with that of humans.

Through integrative multi‐omics profiling of serum metabolites, peripheral immune cells, and gut microbiota, we systematically delineated the molecular and cellular alterations induced by human FMT. Comprehensive transcriptomic and metabolomic analyses revealed widespread reprogramming of gene regulatory networks and significant shifts in immune cell composition, including T cells, monocytes, DCs, and B cells. Notably, we identified subsets of genes displaying human‐like expression patterns in GMH pigs, indicating partial convergence toward human immune and metabolic states.

The use of antibiotic‐treated control pigs maintained under identical environmental and dietary conditions provided rigorous experimental control, enabling confident attribution of observed changes to microbial humanization. While these multi‐omics datasets providing mechanistic insights into the systemic influence of gut microbiota, the current findings remain largely correlative and computational in nature. Although we included selected biochemical assays to support the observed trends, further in vivo functional validation is required to establish causal links between microbial restructuring and host physiological or immunological phenotypes. Future investigation should incorporate targeted perturbation studies to determine the specific roles of human‐derived microbiota taxa in modulating immune function and metabolic homeostasis in this large‐animal model.

## 4 CONCLUSION

We successfully established a microbiota‐humanized pig model by transplanting human fecal microbiota into antibiotic‐treated pigs. This intervention led to substantial remodeling of the host microbial community and serum metabolome, with metabolite profiles shifting toward a human‐like state. Concurrently, single‐cell transcriptomic analysis further revealed that immune cell subset composition and gene expression profiles in peripheral blood were modulated by microbiota humanization, with enhanced signatures of metabolism and biosynthesis observed across multiple immune lineages. Collectively, these findings demonstrate that human gut microbiota can reprogram both systemic metabolism and immune cell function in a large‐animal host. The resulting microbiota‐humanized pig model provides a translationally relevant platform for investigating human microbiota–host interactions, microbiota‐associated diseases, and the evaluation of microbiome‐target therapies. Moreover, this model may hold future utility for xenotransplantation research and preclinical safety assessments.

## 5 METHODS

### Pig and human sample collection

The pig samples used in this study were obtained from the Beijing Farm Animal Research Center. All pigs were reared in accordance with the Guide for the Raising and Use of Laboratory Animals, Institute of Zoology, Chinese Academy of Sciences. Human samples used in this study were obtained from healthy adult individuals. Human and pig information were shown in Table S3. All procedures were approved by the Biomedical Research Ethics Committee of the Institute of Zoology, Chinese Academy of Sciences (1OZ‐IACUC‐2021‐008).

### 5.1 Antibiotic treatment

Pigs were treated with ampicillin (Gold Biotechnology) 60 mg–130 mg/day, vancomycin (Alfa Aesar) 15 mg/day, neomycin (EMD Millipore) 15 mg–30 mg/day, and metronidazole (Alfa Aesar) 7.5 mg/kg [34]. Fresh antibiotic cocktails were prepared every day [26].

### 5.2 Collection and preservation method of human feces

We collected fresh feces samples in the early morning and placed them in sterile centrifuge tubes or other sterile containers. We squeezed the feces into the small grid of the mold (about 2 mL/grid, weighing about 2 g), quickly freezer them in liquid nitrogen for 3–5 min, and store at −80°C.

### 5.3 16S rRNA gene sequencing and analysis

Total genomic DNA from all microbial samples was extracted using the cetyltrimethylammonium bromide/sodium dodecyl sulfate (CTAB/SDS) method. DNA concentration and purity were assessed via electrophoresis on 1% agarose gels. The V3–V4 hypervariable regions of the bacterial 16S rRNA gene were amplified using region‐specific primers 341F (CCTAYGGGRBGCASCAG) and 806R (GGACTACNNGGGTATCTAAT), each tagged with a unique barcode [35]. PCR amplification was carried out using Phusion High‐Fidelity PCR Master Mix (New England Biolabs, USA). PCR products were mixed with 1× loading buffer containing SYBR Green and analyzed on a 2% agarose gel. Samples exhibiting a distinct band at approximately 400–450 bp were selected for downstream processing. Amplicons from different samples were pooled in equimolar concentrations and purified using either the Qiagen Gel Extraction Kit (Qiagen) or the GeneJET Genomic DNA Purification Kit (Thermo Scientific). Sequencing libraries were prepared using the TruSeq DNA PCR‐Free Sample Preparation Kit (Illumina), following the manufacturer's protocol, with unique index codes added to each sample. Library quality and concentration were assessed using a Qubit® 2.0 Fluorometer (Thermo Scientific) and an Agilent 2100 Bioanalyzer system. Paired‐end sequencing was performed on an Illumina HiSeq platform.

Raw sequence data were processed using the QIIME 2 software package (version 2020.2; https://docs.qiime2.org/2020.2/) [36]. After initial quality control, amplicon sequence variants (ASVs) were inferred using the DADA2 algorithm. ASVs detected in fewer than two samples or with total counts below five were excluded to reduce noise. Taxonomic assignment was performed by aligning ASVs to the SILVA reference database (version 138), clustered at 99% sequence identity, using a classifier pre‐trained with QIIME 2 [37]. A feature table of ASVs was generated, and the relative abundance of each taxon was calculated at multiple taxonomic levels and normalized to a total of 100% per sample.

### 5.4 Metagenome sequencing and analysis

The total DNA of RM feces was extracted using Tiangen DNA Stool Mini Kit (TIANGEN Biotech Co., Ltd.) according to the manufacturer's protocol. The extracted DNA was quantified by the NanoDrop. DNA samples with concentration >10 ng/μL and A260/A280 > 1.6 were used for library preparation. The concentration of the final library was >5 ng/μL in a volume of 50 μL. The Novogene Co., Ltd. performed Illumina sequencing.

For metagenomic shotgun sequencing data, low‐quality raw paired‐end reads and adapters were removed using trim‐galore v0.5.0 (q > 20) and cut‐adapter v1.11 [38]. Filtered high‐quality reads were aligned to the human and pig genomes using Bowtie2 v2.1.0 [39] for de‐human and de‐pig contamination. Taxonomic profiling of the cleaned metagenomic reads was carried out using Kraken2 v2.1.3 [40] based on nr database. Relative abundance of the identified taxa was determined for each sample, and normalized to a total abundance of 100% at the different levels, respectively.

### 5.5 scRNA‐seq library generation

Pig blood was collected from the anterior vena cava. PBMCs were isolated by Ficoll gradient centrifugation. Single cells were suspended in PBS containing 0.04% BSA. About 6,000 cells were added to each channel, and the target cell was estimated to be about 3,000 cells. Captured cells were lysed, and the released RNA was barcoded through reverse transcription in individual GEMs. Reverse transcription was performed on a S1000TM Touch Thermal Cycler (Bio‐Rad) at 53°C for 45 min, followed by 85°C for 5 min, and held at 4°C. The cDNA was generated and then amplified, and quality assessed using an Agilent 4200(performed by CapitalBio Technology). Single‐cell RNA‐Seq library preparation According to the manufacturer's introduction, Single‐cell RNA‐seq libraries were constructed using Single Cell 3'Library and Gel Bead Kit V3.1 (10× Genomics, 1000075). The libraries were finally sequenced using an IlluminaNovaseq. 6000 sequencer with a sequencing depth of at least 100,000 reads per cell with pair‐end 150 bp (PE150) reading strategy (performed by CapitalBio Technology).

### 5.6 scRNA‐seq data preprocessing

The Cell Ranger software was obtained from the 10× Genomics website. Raw sequencing data were converted to FASTQ format using Cell Ranger RNA mkfastq [41]. Alignment of scRNA‐seq analyses was completed using the 10× Genomics Cell Ranger RNA pipeline (version 1.2.0). The pig sample library was aligned to an indexed *Sus scrofa*, Sscrofa10.2 genome using CellRanger‐count (3.0.1). At the same time, we used the homologous genes of humans and pigs published in our previous articles as gene features (14377 genes).

We read the data matrix output by cellranger into R (v4.0.3) and processed it using Seurat (v4.0.1). First, we set the parameters min. cells = 3, min. features = 200 to filter the cells. After filtering, the number of cells obtained was: human1 18,289, human2 18,569, Pig1 12,476, Pig2 12,609, humanized Pig1 12,258, and humanized Pig2 12,466. Then the percentage of mitochondrial (“percent.mito”) and ribosome (“percent.ribo”) related genes of each cell was calculated. We set the parameters nFeature_RNA > 200 & nFeature_RNA < 3000 & percent.mt < 20 & nCount_RNA > 500 & nCount_RNA < 15000 to filter the remaining cells. After filtering, the number of remaining sample cells can be found in Table S4.

### 5.7 Enrichment analysis

GO enrichment, KEGG enrichment, reactome enrichment and Disease enrichment (human only) of cluster markers were performed using KOBAS software (http://kobas.cbi.pku.edu.cn/ ) [42], using humanized genes of each cluster. The results were visualized using R package. Protein–protein interaction was obtained from the STRING database with a combined score ≥400 (https://string-db.org/) and visualized using Cytoscape (3.6.0) [43, 44]. GSEA was performed by using GSEA software version 2.2.2.4 [45, 46].

### 5.8 Quasi‐targeted metabolomics

Human, pig, and humanized pig plasma samples were collected and sent to Novogene Co., Ltd for processing and analysis. The samples (100 μL) were placed in the EP tubes and resuspended with prechilled 80% methanol by well vortex. Then the samples were incubated on ice for 5 min and centrifuged at 15,000 *g*, 4°C for 20 min. Some of the supernatant was diluted to a final concentration containing 53% methanol with LC‐MS grade water. The samples were subsequently transferred to a fresh Eppendorf tube and then centrifuged at 15,000 *g*, 4°C for 20 min. Finally, the supernatant was injected into the LC‐MS/MS system for analysis [47, 48].

The metabolites were annotated using the KEGG database (http://www.genome.jp/kegg/), HMDB database (http://www.hmdb.ca/) and Lipidmaps database (http://www.lipidmaps.org/).

### 5.9 Statistical analysis

The results are presented as means ± SD. An unpaired student's *t*‐test was used to test differences between the two groups, as based on the assessment of data variance. Data were examined using one‐way ANOVA, with Bonferroni correction for multiple comparisons. All analyses were performed using GraphPad Prism software (version 8). *p < 0.05, **p < 0.01, and ***p < 0.001. Alpha diversity was assessed by calculating the Richness, using the vegan R package. Bray–Curtis distances were calculated using the vegdist function from the vegan R package. Microbiota community‐level differences were assessed by Permutational Multivariate Analysis of Variance (PERMANOVA) based on the Relative abundance table, using the adonis function from the vegan R package.

## AUTHOR CONTRIBUTIONS

**Zhaoqi Zhang**: Writing—original draft; conceptualization; investigation; software. **Yanan Xu**: Investigation. **Kun Pang**: Software. **Changhong Wu**: Investigation; formal analysis; visualization. **Chenxu Zhao**: Investigation; writing—original draft; methodology; visualization. **Tong Lei**: Methodology; investigation. **Jiayu Zhang**: Investigation; methodology. **Tang Hai**: Writing—review and editing; visualization; validation. **Fangqing Zhao**: Visualization; writing—review and editing; validation. **Yong Zhao**: Validation; visualization; writing—review and editing.

## CONFLICT OF INTEREST STATEMENT

The authors declare no conflicts of interest.

## ETHICS STATEMENT

All pigs were raised under the Guidelines for the Care and Use of Laboratory Animals Committee of the Institute of Zoology, Chinese Academy of Sciences. Pigs were raised at the Beijing Farm Animal Research Center. The use of human and pig samples was approved by the Institute of Zoology, Chinese Academy of Sciences (IOZ‐IACUC‐2021‐008).

## Supporting information


**Figure S1.** Statistical chart of gut flora richness analysis using Unweighted_unifrac methods.
**Figure S2.** Composition changes of the gut microbiome in the human feces‐transplanted pigs at class‐level.
**Figure S3.** Composition changes of the gut microbiome in the human feces‐transplanted pigs at class‐level in human, pig and Human feces‐transplanted pig.
**Figure S4.** Statistics of physiological indicators of pigs and human feces‐transplanted pigs.
**Figure S5.** Metabolite statistics of serum metabolomics.
**Figure S6.** Functional enrichment of serum differential metabolites.
**Figure S7.** Network of humanized metabolites.
**Figure S8.** Functional enrichment of serum humanized metabolites.
**Figure S9.** Analysis of serum biochemical indicators of humans, pigs and Human feces‐transplanted pigs.
**Figure S10.** Metabolome analysis of control pig and human feces‐transplanted pig tissues.
**Figure S11.** Proteomic analysis of humans, control pigs, and human feces‐transplanted pigs.
**Figure S12.** Single cell quality control of integrated human‐pig‐humanized pig data.
**Figure S13.** Integrated scRNA‐seq populations from human, pig and humanized pig cells.
**Figure S14.** Marker gene expression in PBMCs populations defined from human‐pig‐humanized pig integrated scRNA‐seq.
**Figure S15.** Integrated scRNA‐seq populations from human, pig and humanized pig PBMC cells.
**Figure S16.** Heatmap of top 10 marker gene expression in PBMC populations.
**Figure S17.** Integrated scRNA‐seq populations from human, pig and humanized pig T cells.
**Figure S18.** Marker gene expression in γδT cell populations defined from integrated human‐pig‐humanized pig scRNA‐seq.
**Figure S19.** Heatmap of top 10 marker gene expression in T cell populations.
**Figure S20.** Functional analysis of γδ T cells from integrated human‐pig‐humanized pig data.
**Figure S21.** Integrated scRNA‐seq populations from human, pig and humanized pig myeloid cells.
**Figure S22.** Heatmap of top 10 marker gene expression in myeloid cell populations.
**Figure S23.** Marker gene expression in B cell populations defined from human‐pig‐humanized pig integrated scRNA‐seq.
**Figure S24.** Heatmap of top 10 marker gene expression in B cell populations.

**Table S1.** Standards of fecal donation volunteers.
**Table S2.** Feces feeding time‐dose table for each human microbiota‐transplanted and control pig.
**Table S3.** Basic information for human and pig samples.
**Table S4.** The statistics table of Weighted_unifrac distance.
**Table S5.** Cell counts of each group.
**Table S6.** Maker genes for different PBMCs' clusters.
**Table S7.** Marker genes for T cell clusters.
**Table S8.** Genes in myeloid cell clusters.
**Table S9.** Down‐regulated synapse‐related pathways.
**Table S10.** Marker genes in B cell clusters.

## Data Availability

The human PBMC RNA sequencing datasets generated in this study can be found in the RNA‐Seq data: Gene Expression Omnibus (GSE220297, for information on GEO linking and citing, please refer to: https://www.ncbi.nlm.nih.gov/geo/query/acc.cgi?acc=GSE220297). The pig scRNA‐seq data reported in this paper have been deposited in the OMIX, China National Center for Bioinformation/Beijing Institute of Genomics, Chinese Academy of Sciences (OMIX007789, https://ngdc.cncb.ac.cn/omix/release/OMIX007789; OMIX007790, https://ngdc.cncb.ac.cn/omix/release/OMIX007790; OMIX007791, https://ngdc.cncb.ac.cn/omix/release/OMIX007791; OMIX007792, https://ngdc.cncb.ac.cn/omix/release/OMIX007792). The data and scripts for analysis can be found at: https://github.com/zzqsherry/The-human-like-shifted-metabolomics-and-immune-cell-transcriptomics-in-gut-microbiota-humanized-pigs. Supplementary materials (figures, tables, graphical abstract, slides, videos, Chinese translated version and update materials) may be found in the online DOI or iMeta Science http://www.imeta.science/imetaomics/.
