# Supplementary material for: Microbiota humanization drives human‐like metabolic and immune transcriptomic shifts in pigs
Source: IMetaOmics. 2025 Jun 24;2(3):e70034. doi: 10.1002/imo2.70034 (PMC12806034; doi:10.1002/imo2.70034)
Supplement: Supplementary file 1 — Figure S1. Statistical chart of gut flora richness analysis using Unweighted_unifrac methods. Figure S2. Composition changes of the gut microbiome in the human feces‐transplanted pigs at class‐level. Figure S3. Composition changes of the gut microbiome in the human feces‐transplanted pigs at class‐level in human, pig and Human feces‐transplanted pig. Figure S4. Statistics of physiological indicators of pigs and human feces‐transplanted pigs. Figure S5. Metabolite statistics of serum metabolomics. Figure S6. Functional enrichment of serum differential metabolites. Figure S7. Network of humanized metabolites. Figure S8. Functional enrichment of serum humanized metabolites. Figure S9. Analysis of serum biochemical indicators of humans, pigs and Human feces‐transplanted pigs. Figure S10. Metabolome analysis of control pig and human feces‐transplanted pig tissues. Figure S11. Proteomic analysis of humans, control pigs, and human feces‐transplanted pigs. Figure S12. Single cell quality control of integrated human‐pig‐humanized pig data. Figure S13. Integrated scRNA‐seq populations from human, pig and humanized pig cells. Figure S14. Marker gene expression in PBMCs populations defined from human‐pig‐humanized pig integrated scRNA‐seq. Figure S15. Integrated scRNA‐seq populations from human, pig and humanized pig PBMC cells. Figure S16. Heatmap of top 10 marker gene expression in PBMC populations. Figure S17. Integrated scRNA‐seq populations from human, pig and humanized pig T cells. Figure S18. Marker gene expression in γδT cell populations defined from integrated human‐pig‐humanized pig scRNA‐seq. Figure S19. Heatmap of top 10 marker gene expression in T cell populations. Figure S20. Functional analysis of γδ T cells from integrated human‐pig‐humanized pig data. Figure S21. Integrated scRNA‐seq populations from human, pig and humanized pig myeloid cells. Figure S22. Heatmap of top 10 marker gene expression in myeloid cell populations. Figure S23. Marker gene expression in [file IMO2-2-e70034-s002.docx]

**Supporting Information to**

**Microbiota humanization drives human-like metabolic and immune transcriptomic shifts in pigs**

**Running title:** **Multi-omics analysis of gut microbiota-humanized pigs**

Zhaoqi Zhang^1, 2, 3, 4, #^, Yanan Xu^1, #^, Kun Pang^5, #^, Changhong Wu^1^, Chenxu Zhao^1, 2, 3^, Tong Lei^1^, Jiayu Zhang^1^, Tang Hai^6, 7, 8, *^, Fangqing Zhao^5, *^, Yong Zhao^1, 2, 3, 6, *^

^1^ State Key Laboratory of Membrane Biology, Institute of Zoology, Chinese Academy of Sciences, Beijing, 100101, China; ^2^ CAS Key Laboratory of Quantitative Engineering Biology, Shenzhen Institute of Synthetic Biology, Shenzhen Institute of Advanced Technology, Chinese Academy of Sciences, Shenzhen 518055, China; ^3^ Faculty of Synthetic Biology, Shenzhen University of Advanced Technology, 518107, China; ^4^ Department of Rheumatology and Immunology, Peking University People’s Hospital & Beijing Key Laboratory for Rheumatism Mechanism and Immune Diagnosis (BZ0135), Beijing, 100044, China;^. 5^ Institute of Zoology, Chinese Academy of Sciences, Beijing, 100101, China; ^6^ Beijing Institute for Stem Cell and Regenerative Medicine, Beijing 100101, China; ^7^ Beijing Farm Animal Research Center, Institute of Zoology, Chinese Academy of Sciences, Beijing, 100101, China; ^8^ State Key Laboratory of Stem Cell and Reproductive Biology, Institute of Zoology, Chinese Academy of Sciences, Beijing, 100101, China

^#^ Equal contributions as the first authors

^*^ **Corresponding Authors:**

**Dr.** **Yong Zhao,** Faculty of Synthetic Biology, Shenzhen University of Advanced Technology, 1068 Xueyuan Boulevand, University Town of Shenzhen, Xili Nanshan, Shenzhen 518055, China; CAS Key Laboratory of Quantitative Engineering Biology, Shenzhen Institute of Synthetic Biology, Shenzhen Institute of Advanced Technology, Chinese Academy of Sciences, Shenzhen 518055, China.

**E-mail:** y.zhao1@siat.ac.cn

**Dr. Fangqing Zhao,** Institute of Zoology, Chinese Academy of Sciences, Beichen West Road 1-5, Chaoyang District, Beijing 100101, China.

**E-mail:** zhfq@biols.ac.cn

**Dr. Tang Hai**, Beijing Farm Animal Research Center, Institute of Zoology, Chinese Academy of Sciences, Beijing, 100101, China

**E-mail:** haitang@ioz.ac.cn

**Supplementary figures**


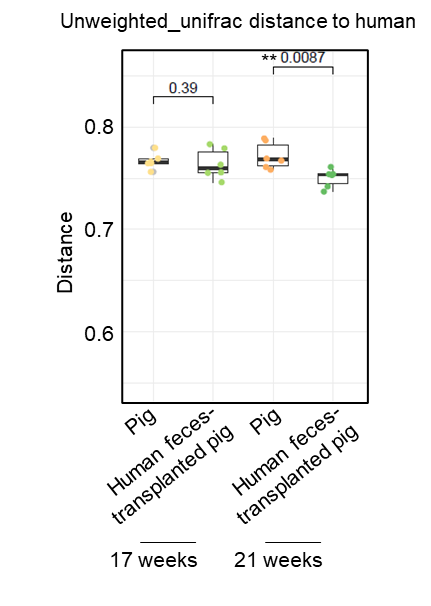


**Figure S1. Statistical chart of gut flora richness analysis using Unweighted_unifrac methods.**

Sample numbers of intestinal flora sequencing: 4 human donor feces samples, 6 control pig samples, and 6 human feces-transplanted pig samples. (**) *p* < 0.01, *t*-test.


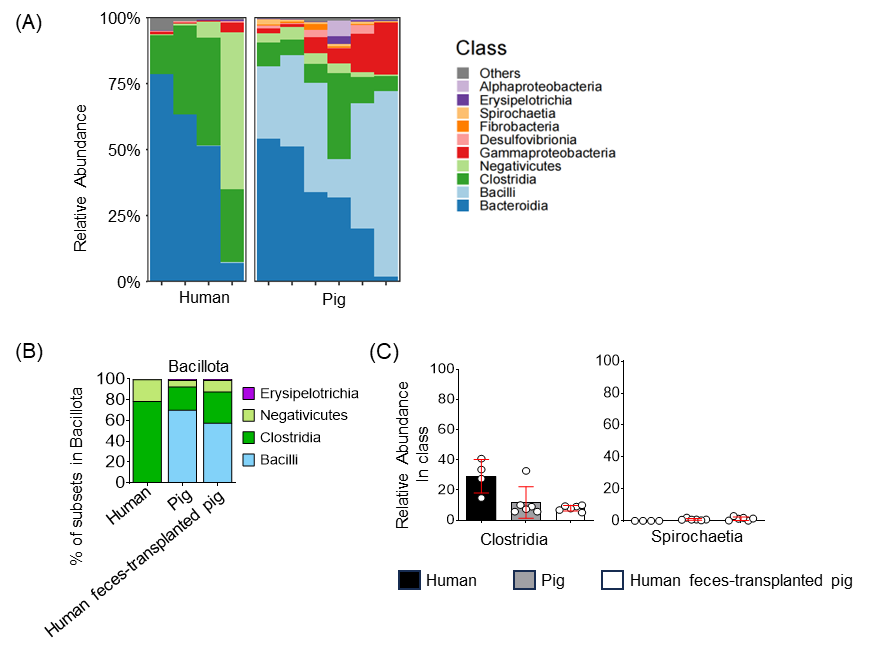


**Figure S2. Composition changes of the gut microbiome in the human feces-transplanted pigs at class-level.**

(A) Class-level sample composition. (B) Statistical chart of class-level intestinal flora with humanization trend. (C) The proportions of Clostridia and Spirochaetia in pigs, human and human feces-transplanted pigs. Number of intestinal flora sequencing samples: 4 human samples (offered feces for transplantation), 6 control pigs, 6 human feces-transplanted pigs. Data in human, Pig, and humanized pig samples are presented as mean ± SD.


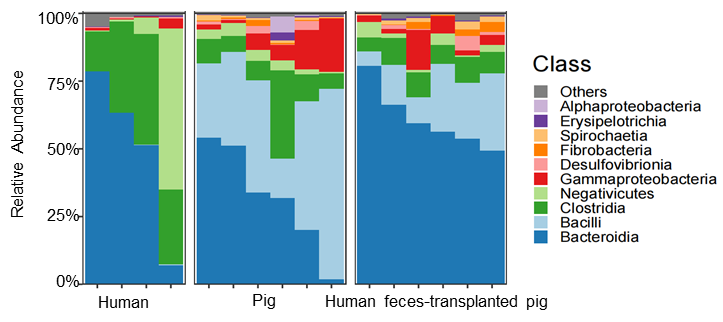


**Figure S3. Composition changes of the gut microbiome in the human feces-transplanted pigs** **at class-level in human, pig and Human feces-transplanted pig.**

Number of intestinal flora sequencing samples: 4 humans (feces donors), 6 control pigs, 6 human feces-transplanted pigs.


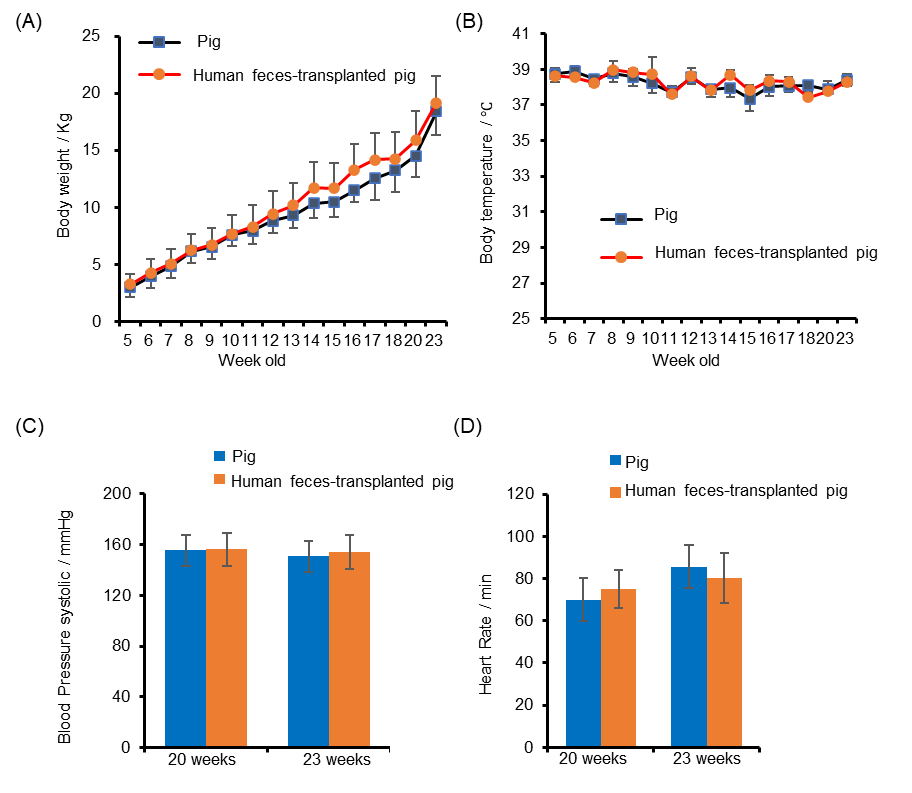


**Figure S4. Statistics of physiological indicators of pigs and human feces-transplanted pigs.**

Line chart of body weight (A) and body temperature (B) of antibiotics-treated control pigs and human feces-transplanted pigs. Blood pressure systolic (C) and heart rate (D) statistics diagram for pigs and human feces-transplanted pigs at 20 and 23 weeks. Sample numbers: 6 control pigs, and 6 human feces-transplanted pigs. Data in human, Pig, and humanized pig samples are presented as mean ± SD.


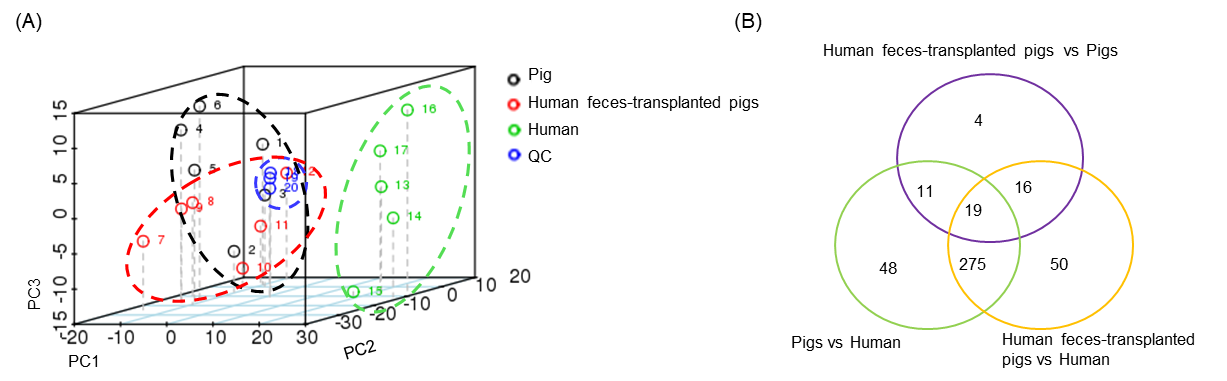


**Figure S5. Metabolite statistics of serum metabolomics.**

(A) PCA plot of serum metabolomics sample distribution. (B) Venn diagram of serum metabolite number statistics. Sample numbers of serum metabolome sequencing: 5 humans, 6 control pigs, and 6 human feces-transplanted pigs.


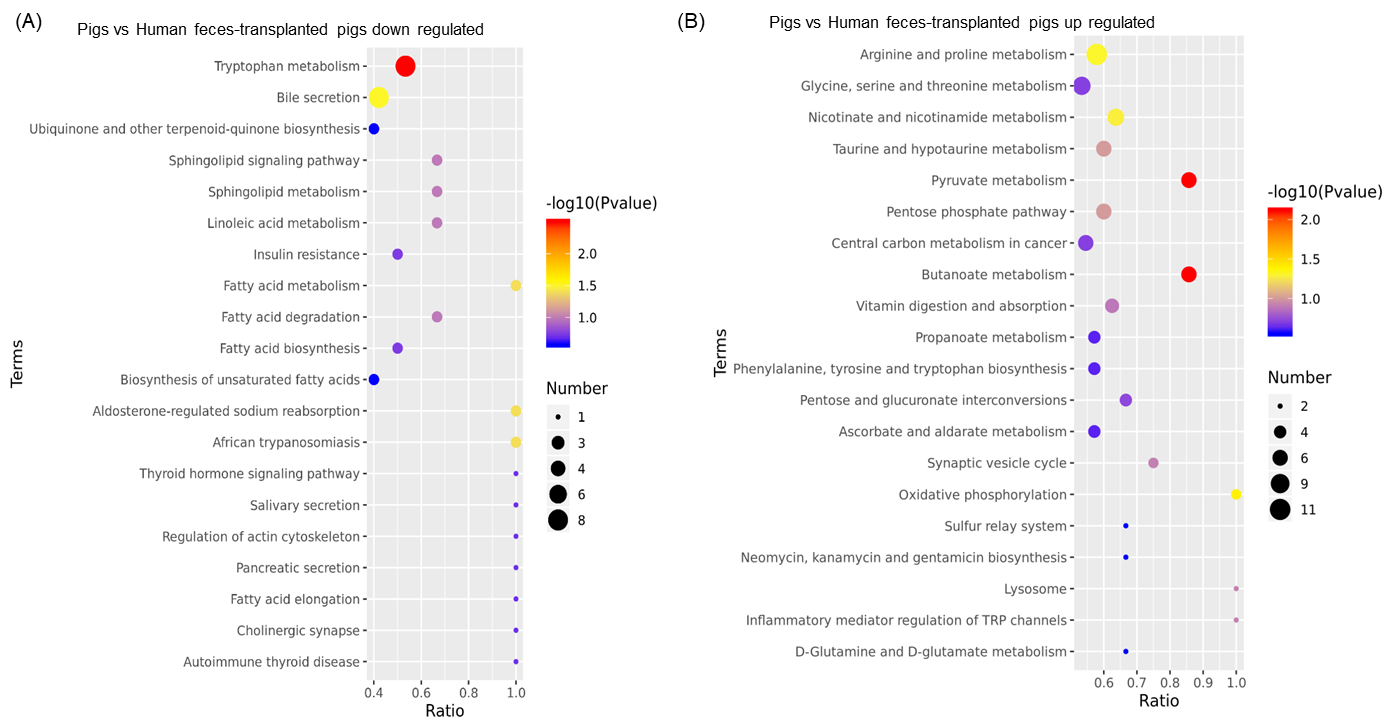


**Figure S6. Functional enrichment of serum differential metabolites.**

KEGG enrichment bubble chart of down-regulated (A) and up-regulated (B) metabolites in control pigs vs humanized pigs. Sample numbers of serum metabolome sequencing: 5 humans, 6 pigs, and 6 human feces-transplanted pigs.


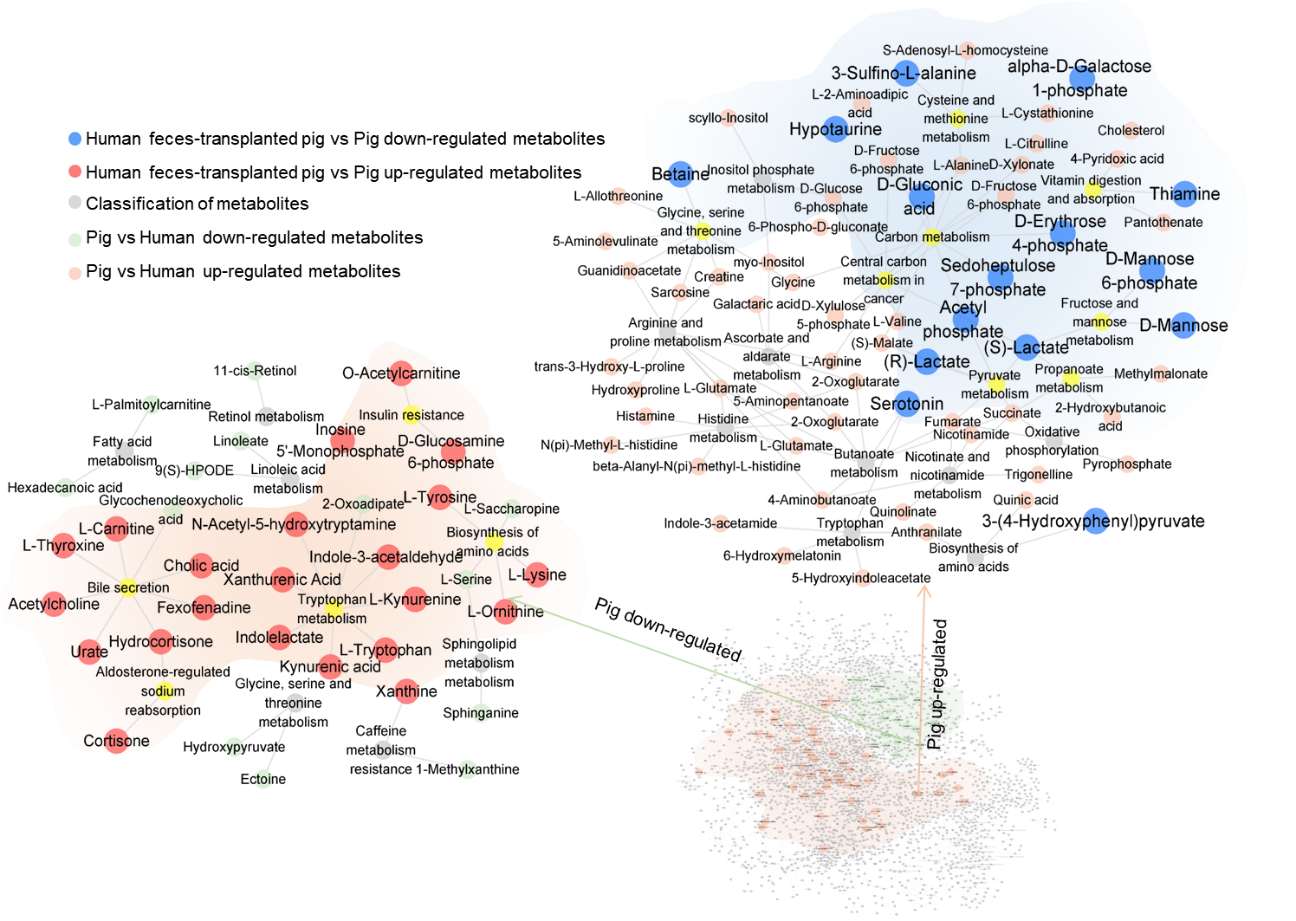


**Figure S7. Network of humanized metabolites.**

Sample numbers of serum metabolome sequencing: 5 humans, 6 pigs, and 6 human feces-transplanted pigs.


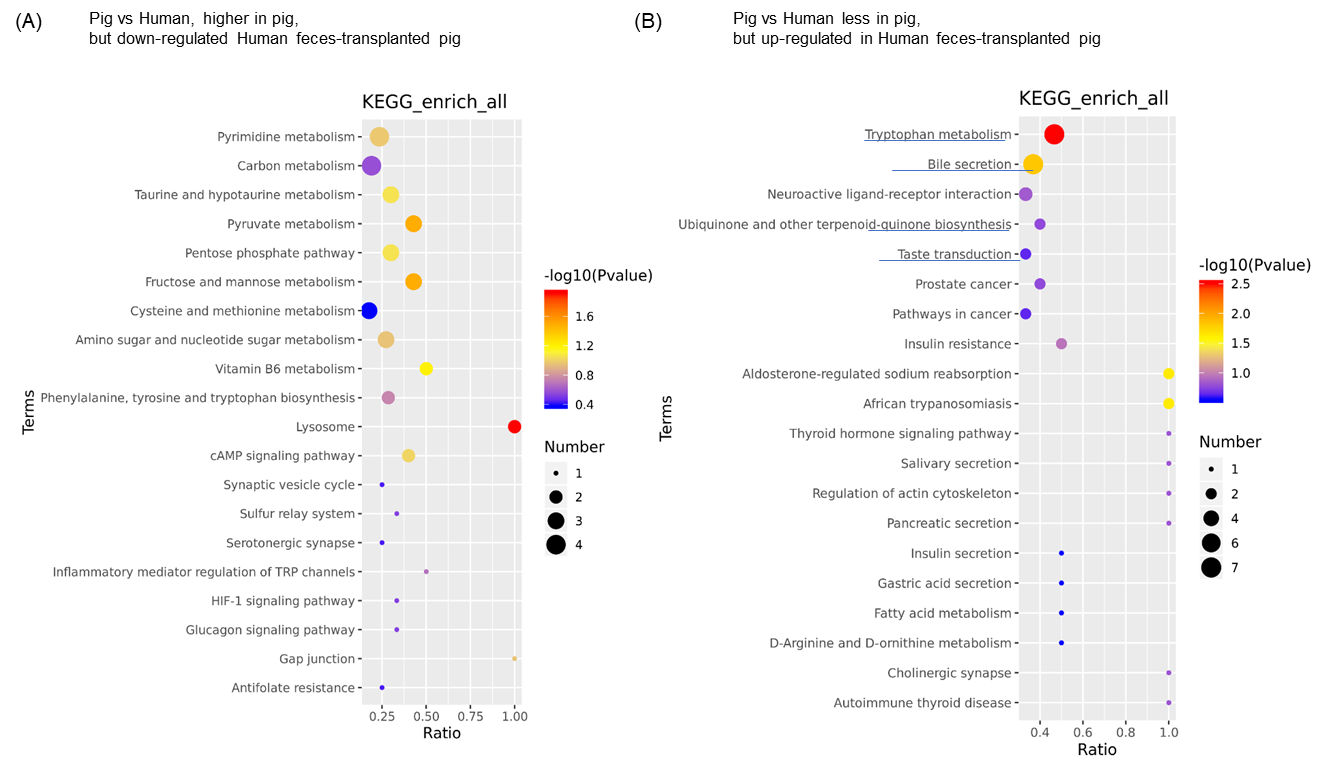


**Figure S8. Functional enrichment of serum humanized metabolites.**

KEGG enrichment bubble chart of up-regulated (A) and down-regulated (B) humanized metabolites in Pig vs Humanized pig. Sample number of serum metabolome sequencing: 5 humans, 6 pigs, and 6 human feces-transplanted pigs.


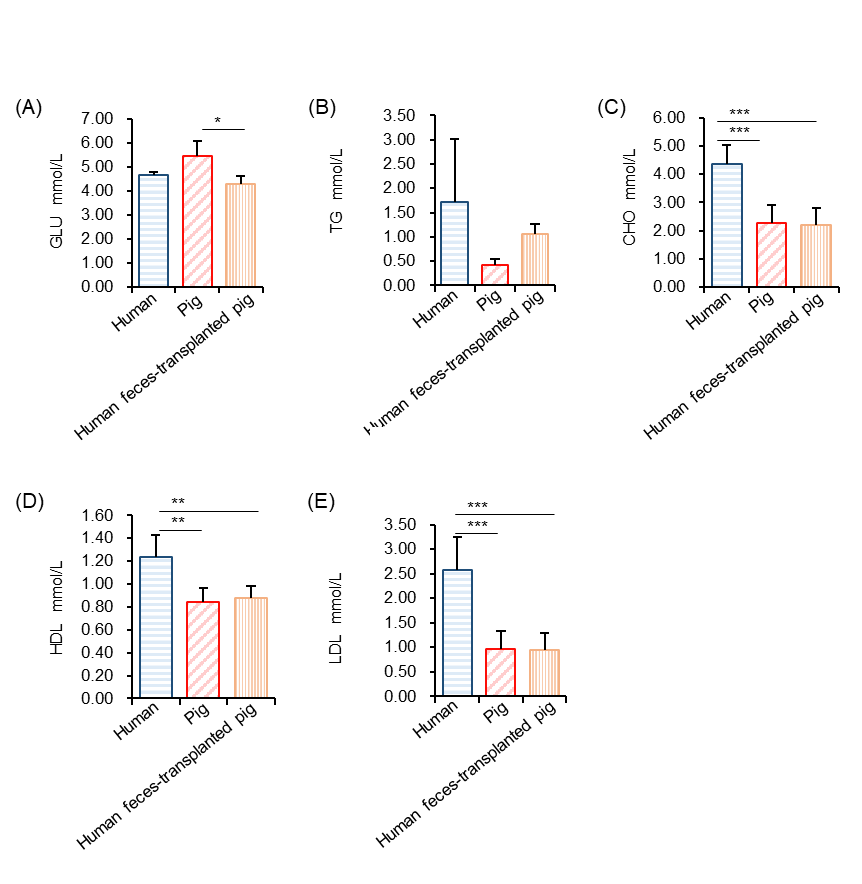


**Figure S9. Analysis of serum biochemical indicators of humans, pigs and Human feces-transplanted pigs.**

GLU (A), TG (B), CHO (C), HDL (D) and LDL (Estatistics diagram forhuman, control pigs and humanized pigs at 27 weeks. Number of pig samples: 6 control pigs, and 6 human feces-transplanted pigs. Data in human, Pig, and humanized pig samples are presented as mean ± SD. (*) *p* < 0.05, (**) *p* < 0.01, (***) *p* < 0.001, data were examined using one-way ANOVA, with Bonferroni correction.


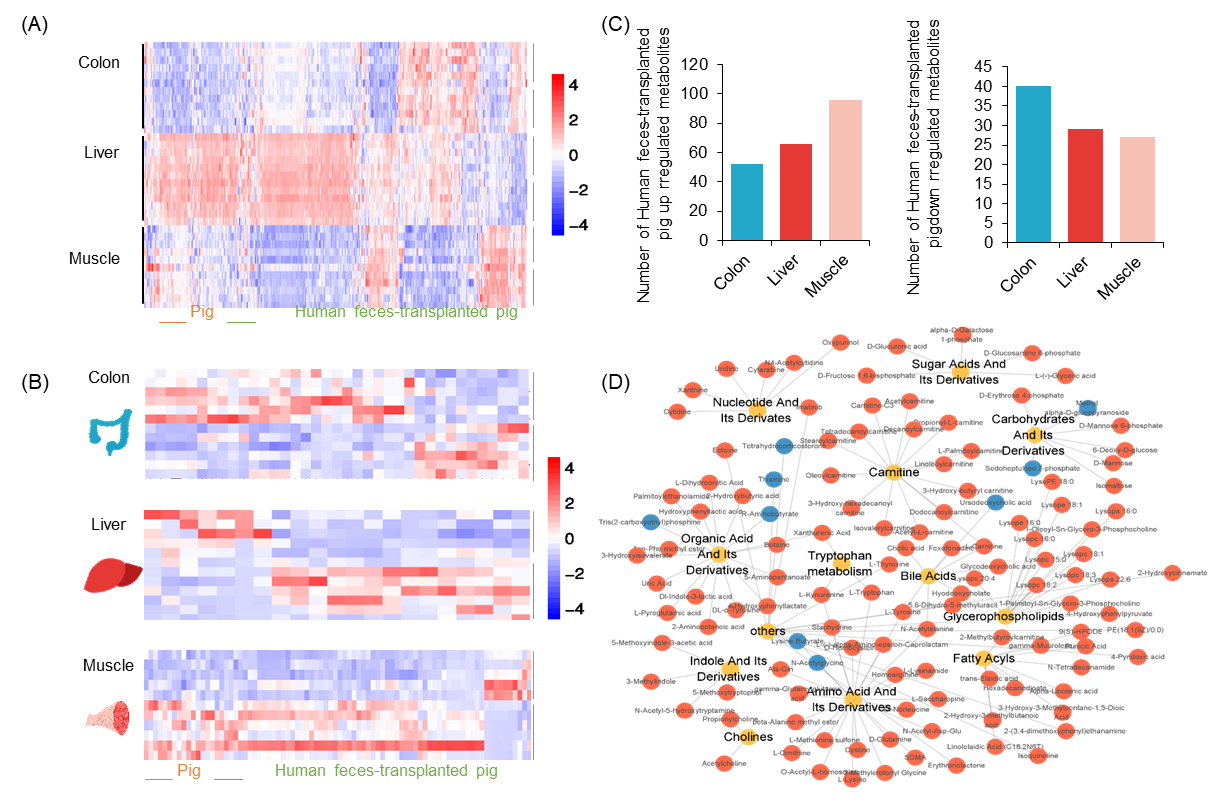


**Figure S10. Metabolome analysis of control pig and human feces-transplanted pig tissues.**

(A) Heatmap of differential metabolites of pig colons, liver and muscle between control pigs and feces-humanized pigs. Heatmap (B) and statistic diagram (C) of human-like changed metabolites of pig colon, liver and muscle. (D) Network of human-like changed metabolites in pig colons, liver and muscle. Sample numbers of metabolome sequencing: 6 control pigs, 6 human feces-transplanted pigs.


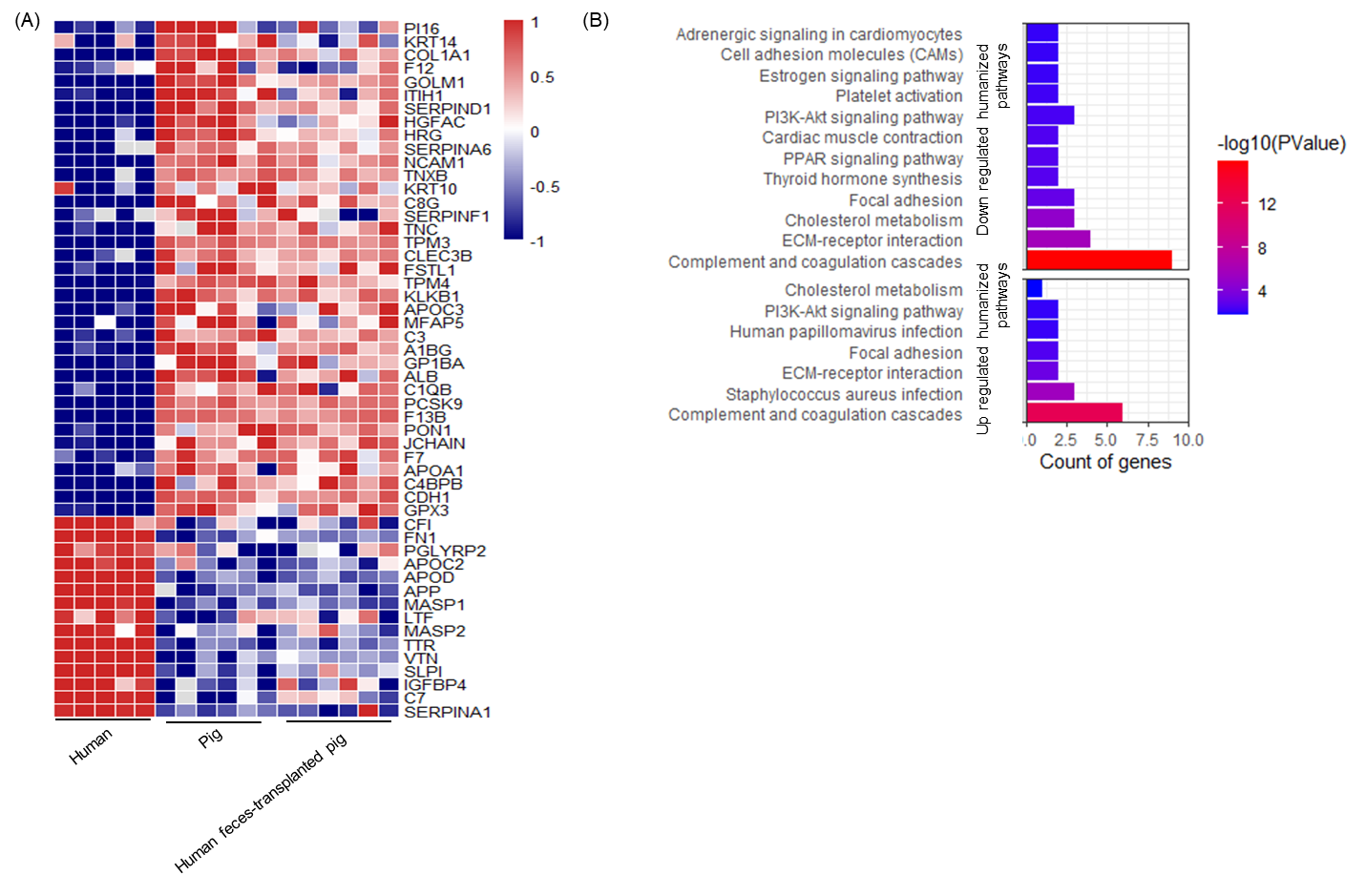


**Figure S11. Proteomic analysis of humans, control pigs, and human feces-transplanted pigs.**

(A) Heatmap of altered protein expression in humans, pigs and humanized pigs. (B) KEGG enrichment statistics of humanized changed protein expressions. Sample numbers of serum protein sequencing: 5 humans, 6 control pigs, and 6 human feces-transplanted pigs.


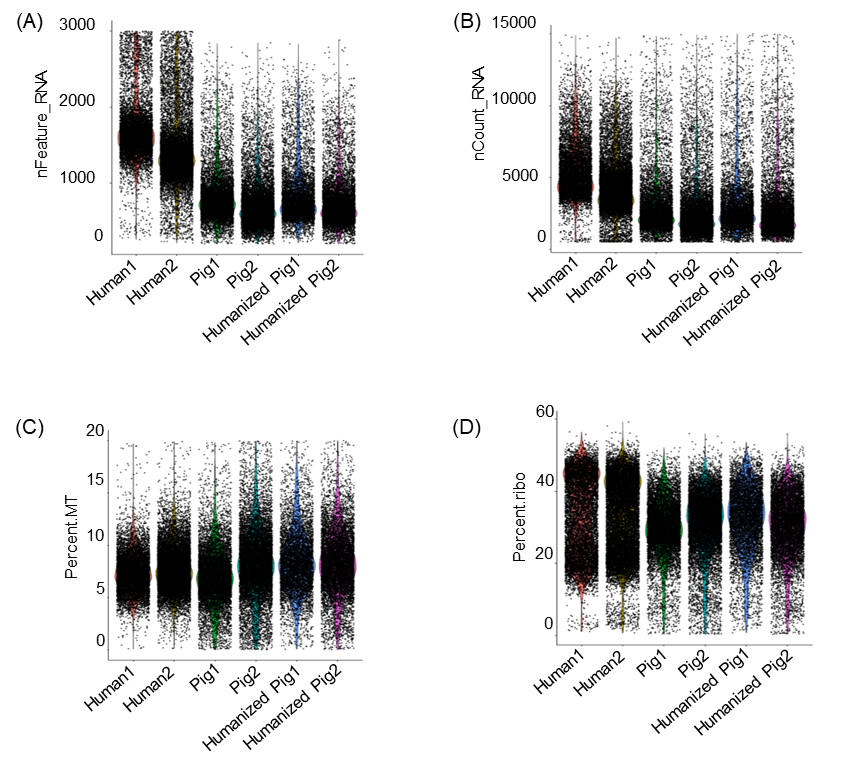


**Figure S12. Single cell quality control of integrated human-pig-humanized pig data.**

Vlnplot of human-pig-humanized pig nFeature_RNA (A), nCount_RNA (B), Percent.MT (C) and Percent.ribo (D). 2 PBMC samples of human, control pigs, and human feces-transplanted pigs were tested for scRNA-seq, respectively.


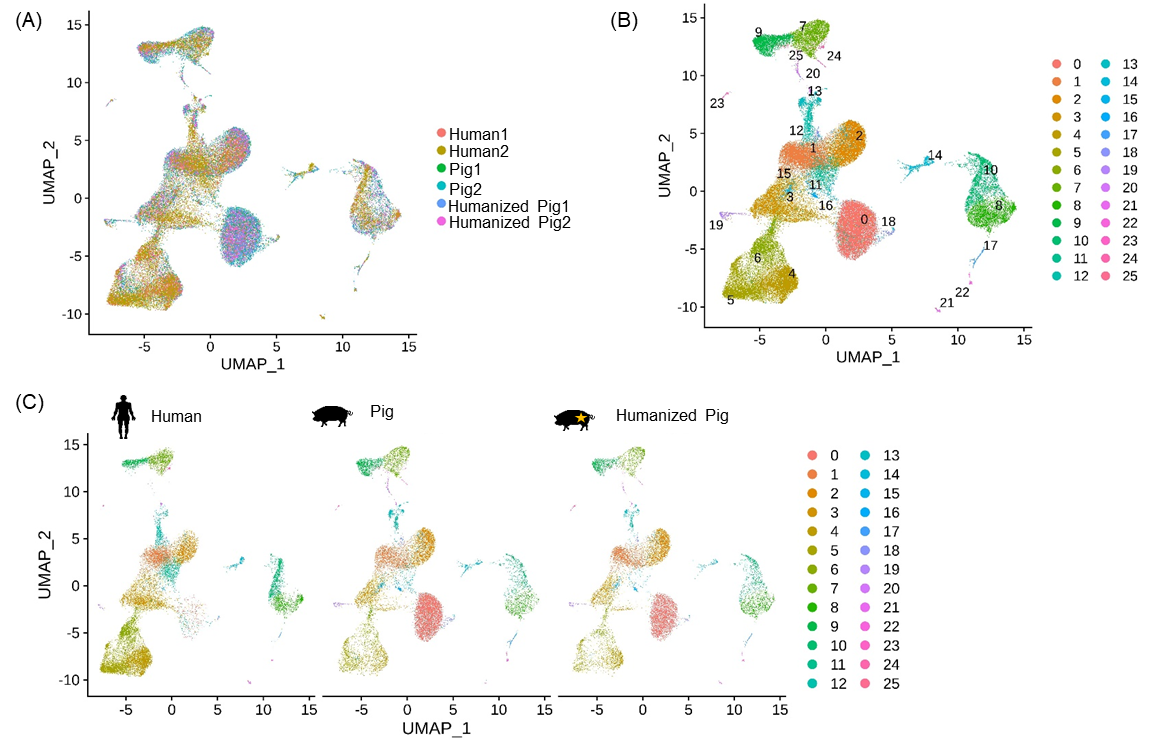


**Figure S13. Integrated scRNA-seq populations from human, pig and humanized pig cells.**

(A) UMAP visualization of human-pig-humanized pig integrated clustering. Each dot represents a cell, which is colored according to sample type. (B) UMAP visualization of human-pig-humanized pig integrated clustering. Each dot represents a cell, which is colored according to cluster type. (C) UMAP visualization of separated human cells, pig cells and humanized pig cells for all cells. Each dot represents a cell, which is colored according to cell type. Number of PBMCs scRNA-seq: 2 humans, 2 control pigs, and 2 human feces-transplanted pigs.


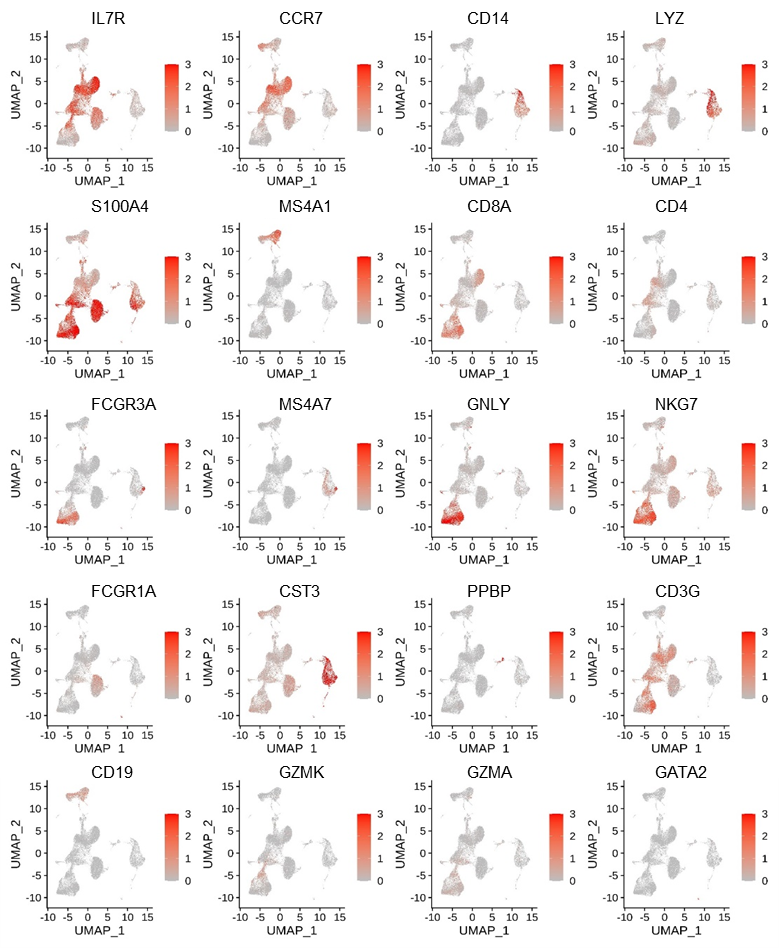


**Figure S14. Marker gene expression in PBMCs populations defined from human-pig-humanized pig integrated scRNA-seq.**

Sample number of PBMCs for scRNA-seq: 2 humans, 2 pigs, and 2 human feces-transplanted pigs.


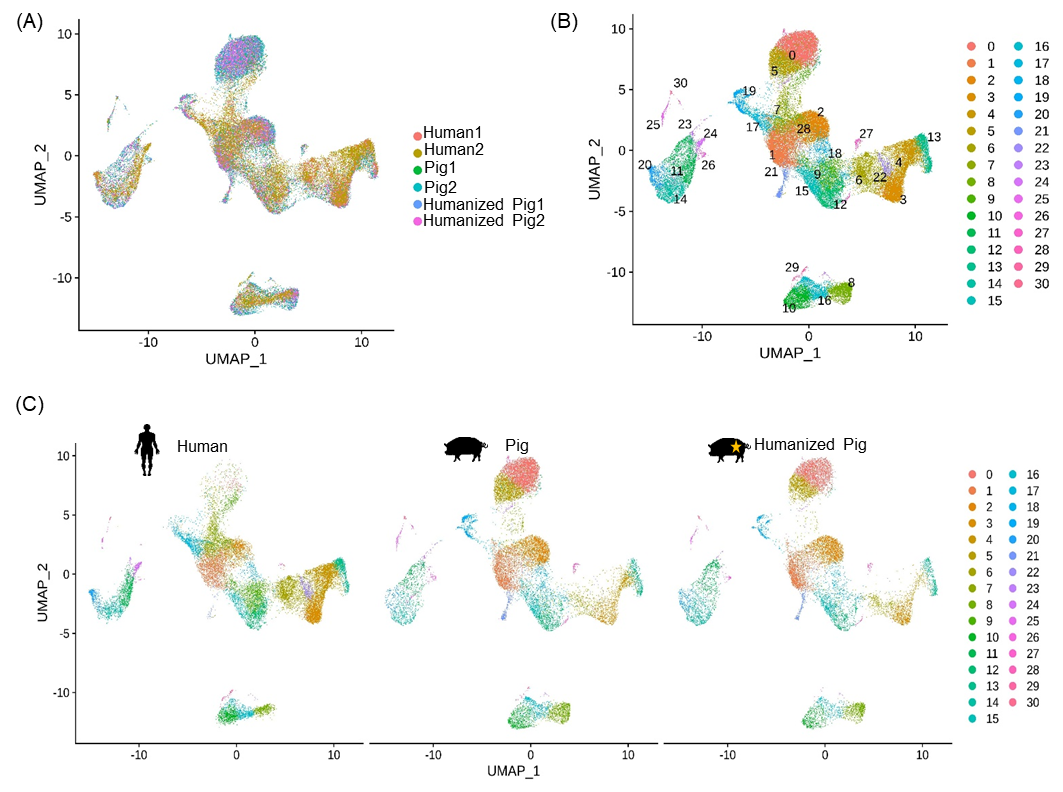


**Figure S15. Integrated scRNA-seq populations from human, pig and humanized pig PBMC cells.**

(A) UMAP visualization of human-pig-humanized pig integrated clustering. Each dot represents a cell, which is colored according to sample type. (B) UMAP visualization of human-pig-humanized pig integrated clustering. Each dot represents a cell, which is colored according to cluster type. (C) UMAP visualization of separated human cells, pig cells and humanized pig cells for all cells. Each dot represents a cell, which is colored according to cell type. Sample number of PBMCs scRNA-seq: 2 humans, 2 pigs, and 2 human feces-transplanted pigs.


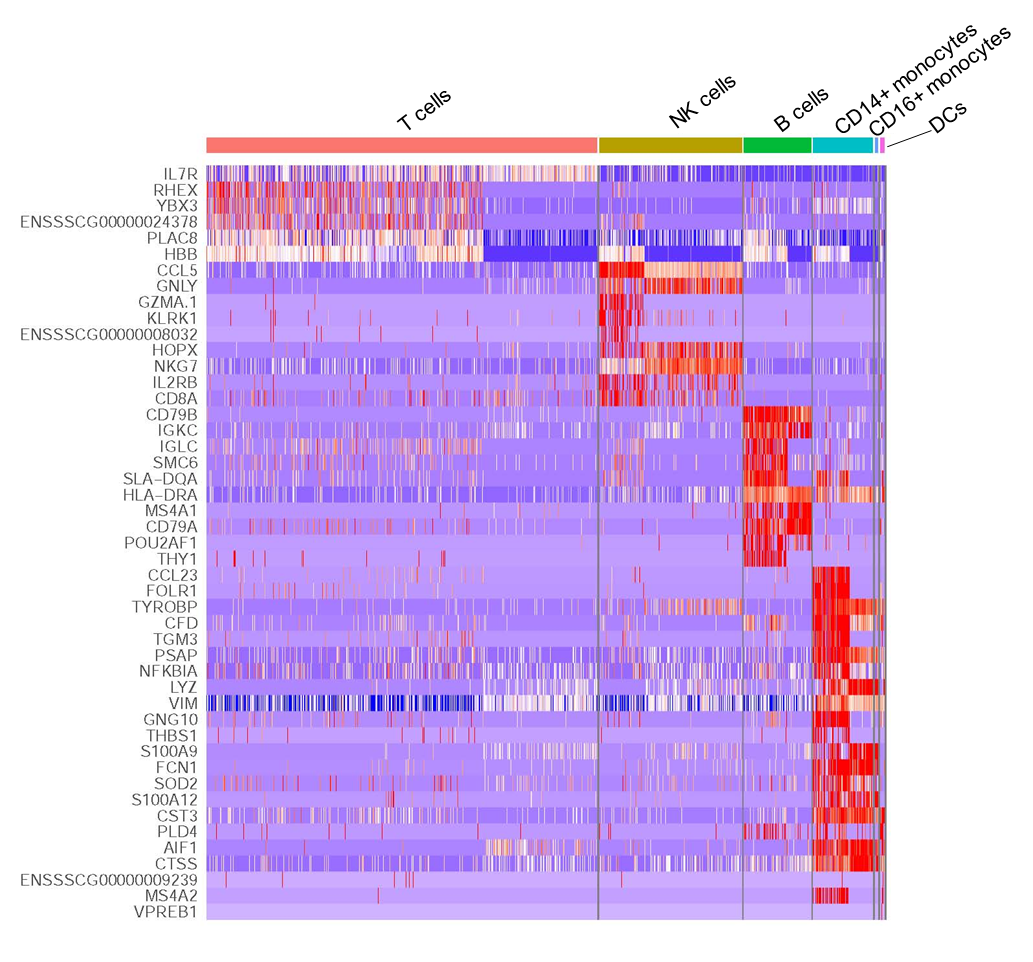


**Figure S16. Heatmap of top 10 marker gene expression in PBMC populations.**

Sample numbers of PBMCs for scRNA-seq: 2 humans, 2 pigs, and 2 human feces-transplanted pigs.


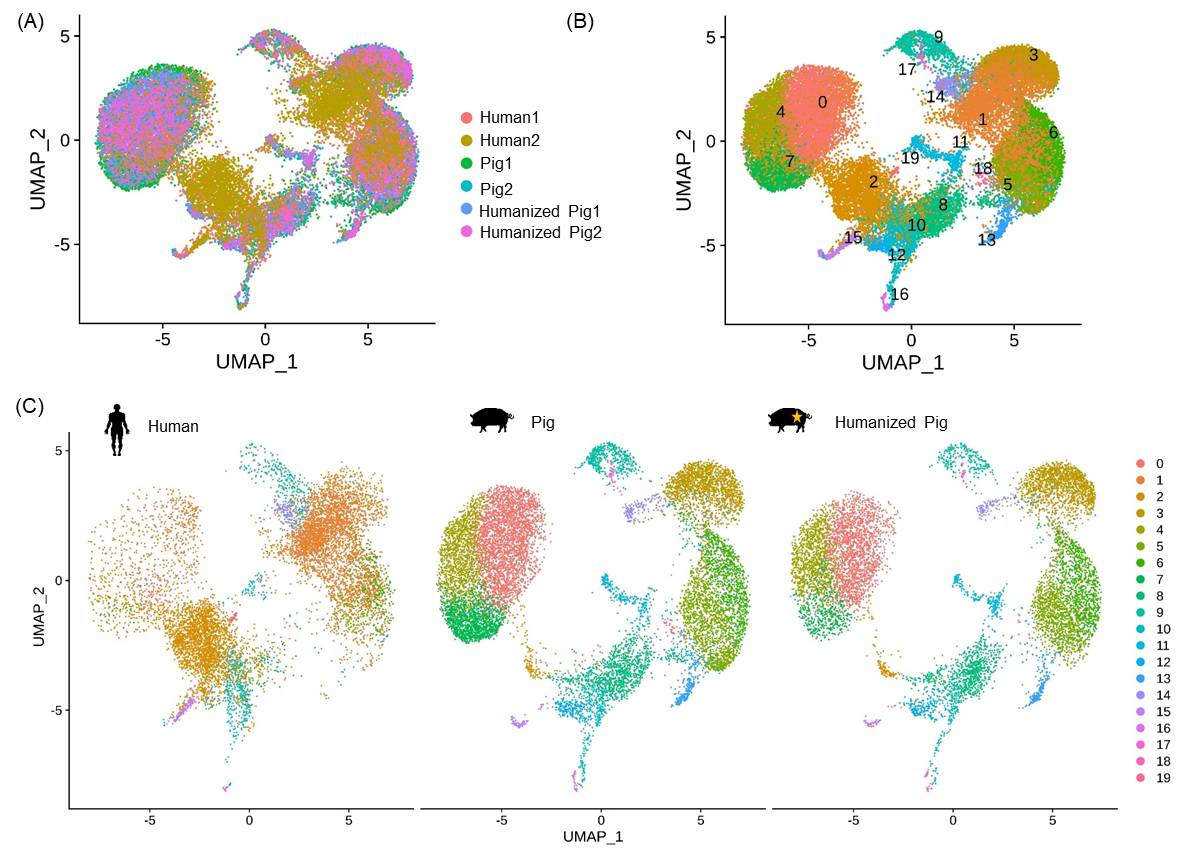


**Figure S17. Integrated scRNA-seq populations from human, pig and humanized pig T cells.**

(A) UMAP visualization of integrated human-pig-humanized pig cell clustering. Each dot represents a cell, which is colored according to sample types. (B) UMAP visualization of integrated human-pig-humanized pig clustering. Each dot represents a cell, which is colored according to cluster types. (C) UMAP visualization of separated cells from human, pigs and humanized pigs. Each dot represents a cell, which is colored according to cell types. Sample numbers of PBMCs for scRNA-seq: 2 humans, 2 pigs, and 2 human feces-transplanted pigs.


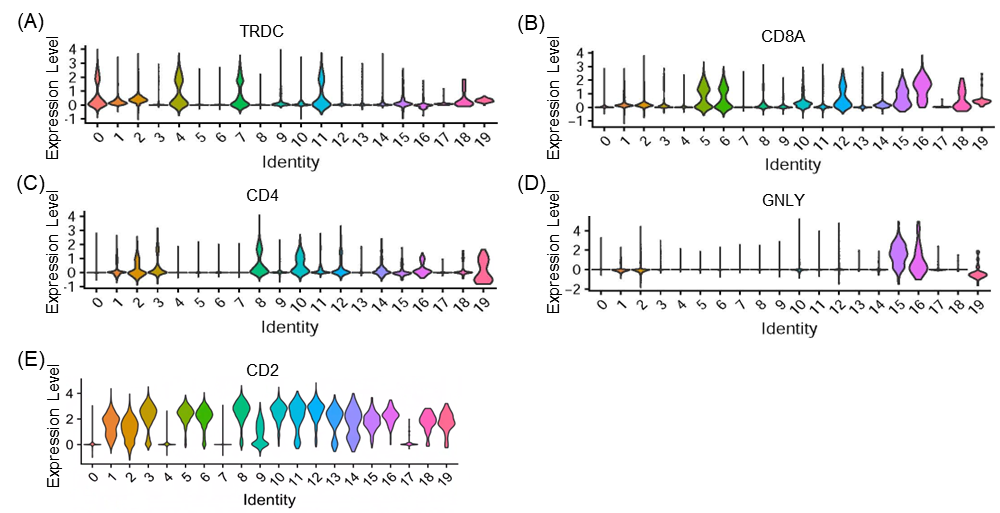


**Figure S18. Marker gene expression in γδT cell populations defined from integrated human-pig-humanized pig scRNA-seq.**

Vlnplot of TRDC (A), CD8A (B), CD4 (C), GNLY (D) and CD2 (E) expression level. Sample numbers of PBMCs for scRNA sequencing: 2 humans, 2 pigs, and 2 human feces-transplanted pigs.


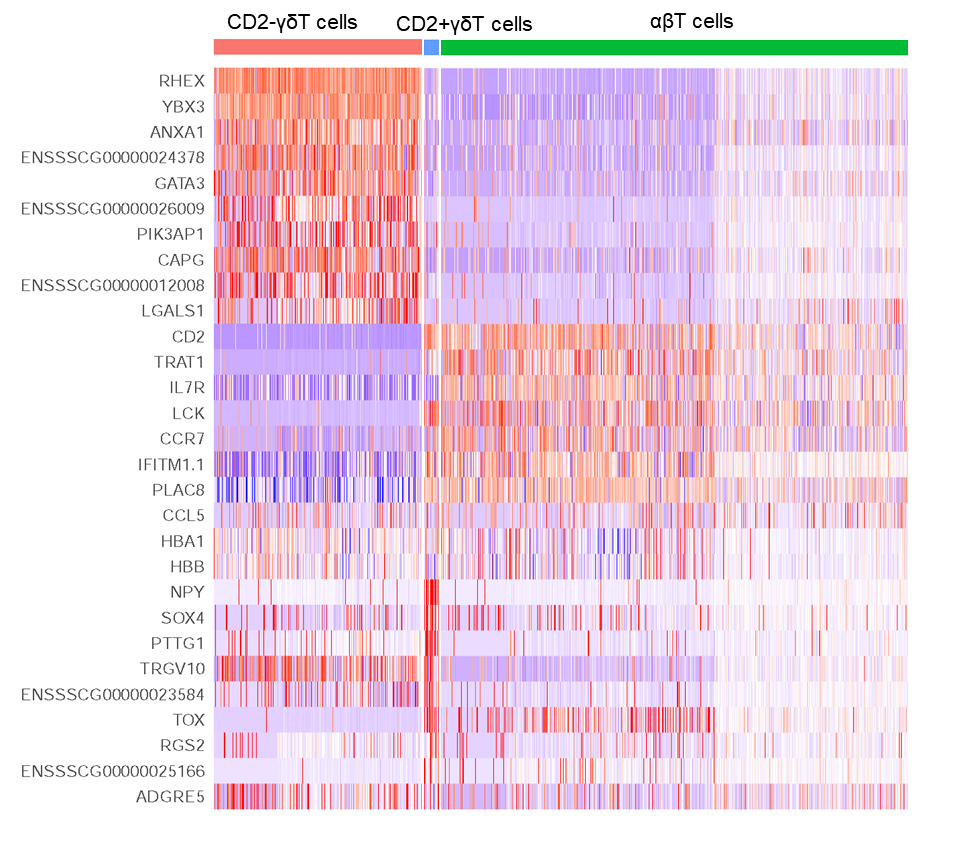


**Figure S19. Heatmap of top 10 marker gene expression in T cell populations.**

Sample numbers of PBMCs for scRNA sequencing: 2 humans, 2 pigs, and 2 human feces-transplanted pigs.


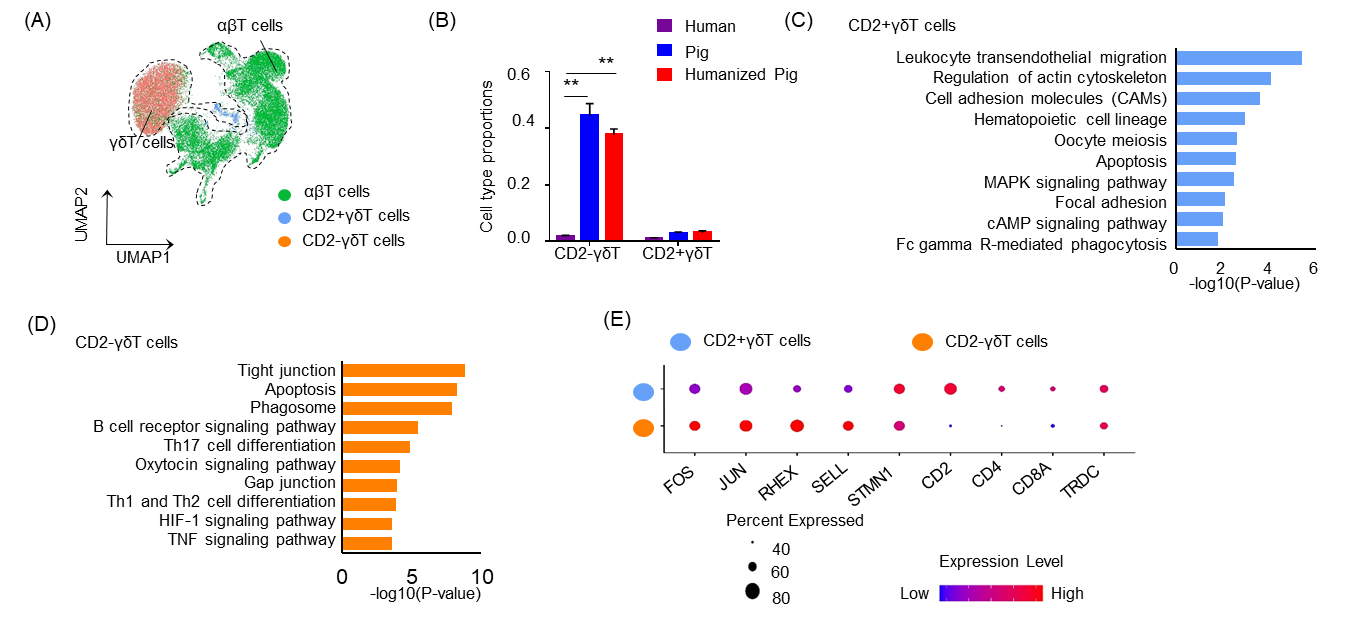


**Figure S20. Functional analysis of γδ T cells from integrated human-pig-humanized pig data.**

(A) UMAP visualization of integrated human-pig-humanized pig T cell clustering. Each dot represents a cell, which is colored according to cluster type. (B) The relative cell proportions (%) of γδT cell subpopulation in human, pigs and humanized pigs, respectively. Statistical diagram of KEGG functional enrichment of characteristic genes in CD2+γδT cells (C) and CD2-γδT cells (D). (E) Bubble chart of highly expressed genes in CD2+γδT cells and CD2-γδT cells. Sample numbers of PBMCs for scRNA sequencing: 2 humans, 2 pigs, 2 human feces-transplanted pigs. Data in human, Pig, and humanized pig samples are presented as mean ± SD. (**) *p* < 0.01, data were examined using one-way ANOVA, with Bonferroni correction.


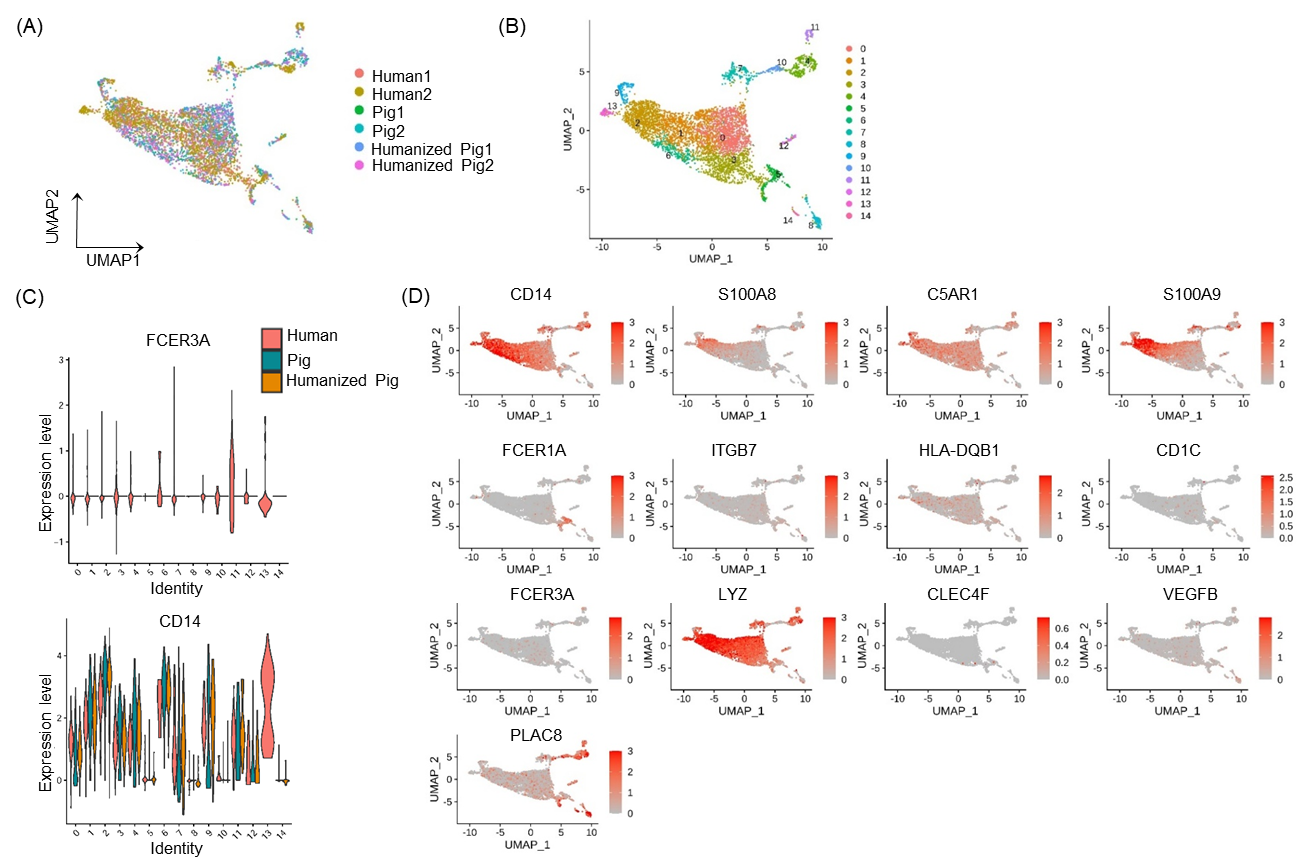


**Figure S21. Integrated scRNA-seq populations from human, pig and humanized pig myeloid cells.**

(A) UMAP visualization of integrated human-pig-humanized pig myeloid clustering. Each dot represents a cell, which is colored according to sample type. (B) UMAP visualization of human-pig-humanized pig integrated clustering. Each dot represents a cell, which is colored according to cluster type. (C) Vlnplot chart of FCGR3A and CD14 expression. (D) Marker gene expression in myeloid populations defined from human-pig-humanized pig integrated scRNA-seq. Sample numbers of PBMCs for scRNA sequencing: 2 humans, 2 pigs, and 2 human feces-transplanted pigs.


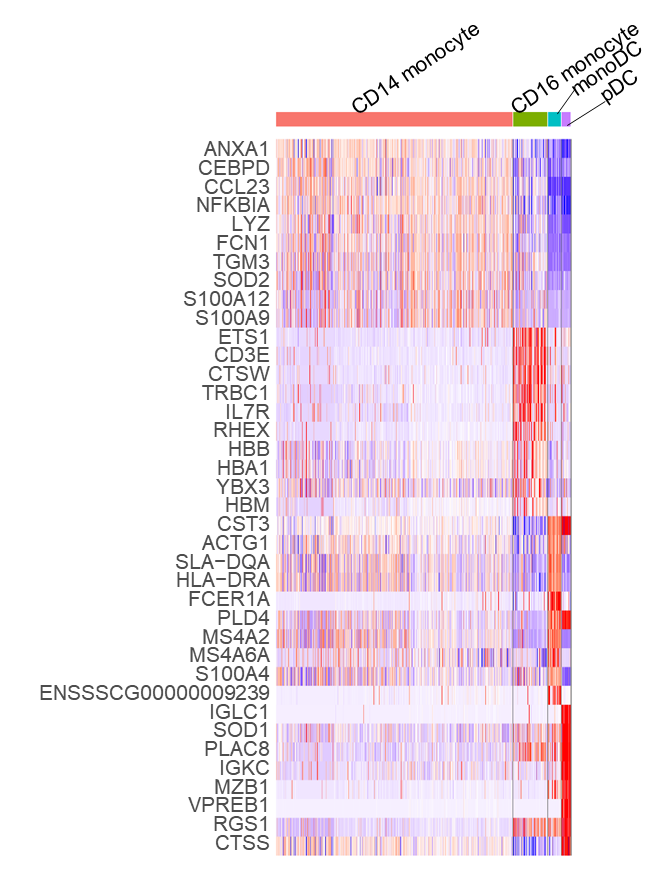


**Figure S22. Heatmap of top 10 marker gene expression in myeloid cell populations.**

Sample numbers of PBMCs for scRNA sequencing: 2 humans, 2 control pigs, and 2 human feces-transplanted pigs.


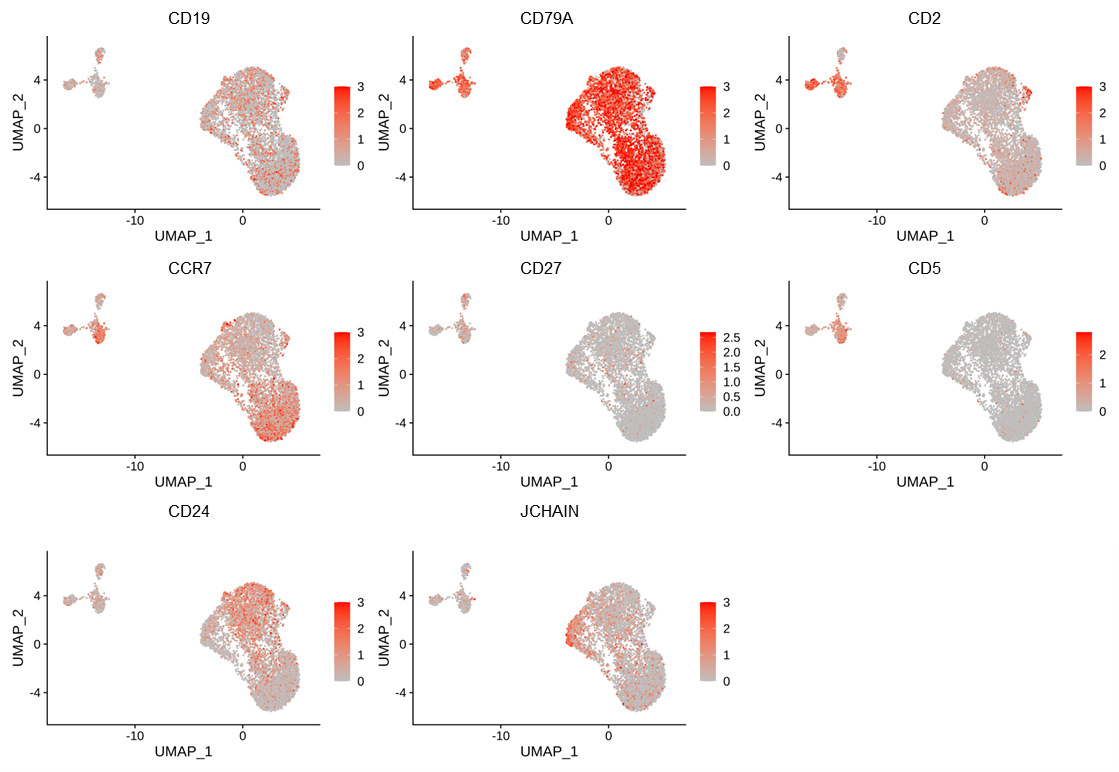


**Figure S23. Marker gene expression in B cell populations defined from human-pig-humanized pig integrated scRNA-seq.**

Sample numbers of PBMCs for scRNA sequencing: 2 humans, 2 control pigs, and 2 human feces-transplanted pigs.


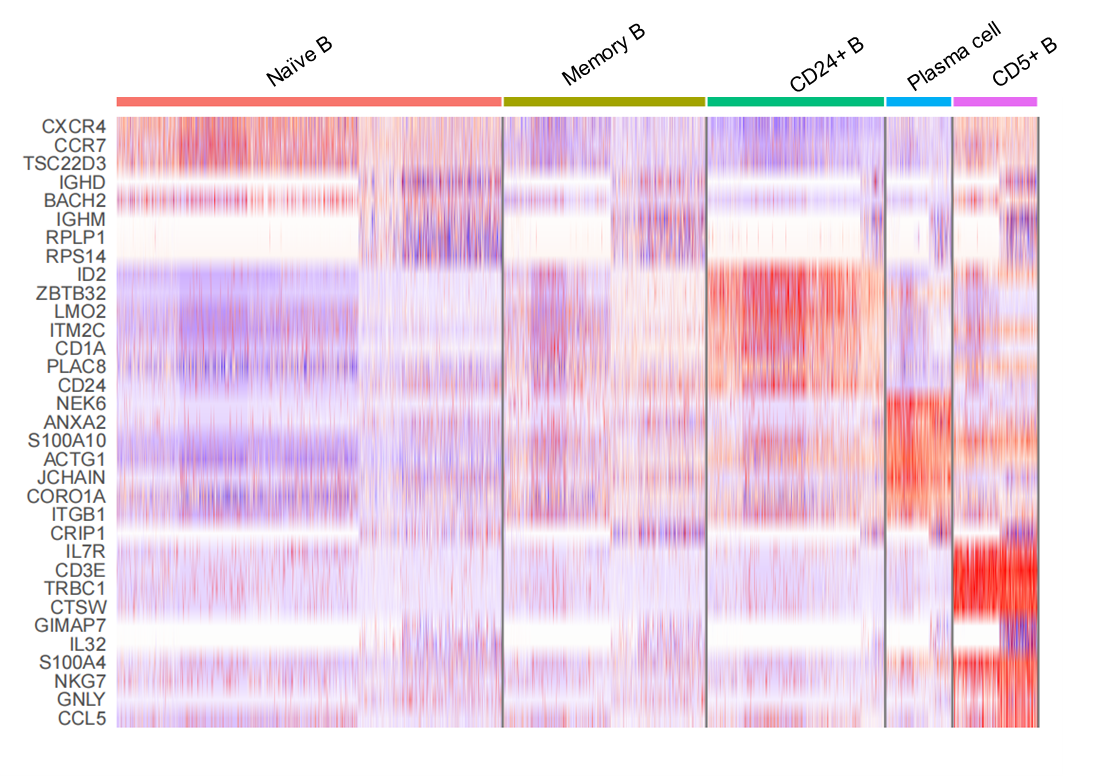


**Figure S24. Heatmap of top 10 marker gene expression in B cell populations.**

Sample numbers of PBMCs for scRNA sequencing: 2 humans, 2 control pigs, and 2 human feces-transplanted pigs.
